# Nanomedicine in Organ Transplantation: From Graft Preservation and Repair to Immunomodulation and Monitoring

**DOI:** 10.7150/thno.128348

**Published:** 2026-01-01

**Authors:** Leyi Wang, Chen Jin, Junjie Zhou, Jiani Yin, Gang Xu, Zhenyu Duan, Jiayin Yang, Qiyong Gong, Kui Luo

**Affiliations:** 1 Department of Radiology, Huaxi MR Research Center (HMRRC), Institution of Radiology and Medical Imaging, Liver Transplant Center, Organ Transplant Center, Department of General Surgery, Frontiers Science Center for Disease-Related Molecular Network, State Key Laboratory of Biotherapy, West China Hospital, Sichuan University, Chengdu 610041, China.; 2 Glasgow College, University of Electronic Science and Technology of China, Chengdu 611731, China.; 3 XiangYa School of Medicine, Central South University, Changsha 410013, China.; 4 Psychoradiology Key Laboratory of Sichuan Province, and Research Unit of Psychoradiology, Chinese Academy of Medical Sciences, Chengdu 610041, China.; 5 Laboratory of Liver Transplantation, Institute of Organ Transplantation, Key Laboratory of Transplant Engineering and Immunology, NHC, West China Hospital of Sichuan University, Chengdu 610041, China.; 6 Xiamen Key Lab of Psychoradiology and Neuromodulation, Department of Radiology, West China Xiamen Hospital of Sichuan University, Xiamen 361021, China.

**Keywords:** organ transplantation, nanomedicine, transplant rejection, ischemia-reperfusion injury

## Abstract

Organ transplantation remains a life-saving intervention for end-stage organ failure. However, its long-term success has been constrained by a few critical challenges, including few noninvasive diagnostic technologies for graft assessment, a lack of effective organ preservation and rewarming techniques to mitigate ischemic damage, the issue of ischemia-reperfusion injury (IRI), the risk of immune-mediated rejection and the requirement of advanced postoperative management. Nanomedicine has been explored for overcoming these challenges for organ transplantation. A myriad of polymeric, inorganic and hybrid nanocarriers have been employed for nanomedicine. Targeting and stimuli-responsive nanomedicine has been developed to improve drug distribution and enhance its therapeutic/diagnostic efficacy. Nanomedicine has been applied for rewarming of large-sized organs, IRI mitigation, immunomodulation, and real-time monitoring. This review examines the mechanisms, elaborates design principles, and covers the application of nanomedicine in organ transplantation at stages of pre- to post-transplantation. The challenges in clinical translation of nanomedicine are discussed and future research directions are proposed. This review will provide a consolidated framework for the development and application of nanomedicine for organ transplantation, ultimately improving the quality of life of transplant recipients.

## 1. Introduction

Organ transplantation is a revolutionary treatment by itself that has significantly enhanced the lives and survival of a patient and continues to serve as an effective treatment to most of the terminal diseases 1. Nonetheless, there are some challenges encountered in this area. The most urgent is the lack of organs donors in the world, as it make many patients live with life threatening complication in expectation of transplantation 2. The risks of posttransplant rejection and complications are high including acute and chronic rejection, graft dysfunction, infection, and cancer all of which could not allow long-term survival of patients 3. There were also some few organs (e.g., the lung, heart, liver, and kidney) clinically transplanted, and also the individual organs pose their own immunological problems. Liver is more immunologically tolerant compared to other ones 4, but transplantation in lungs, and islet cells usually produces intense immune response and auto immunity, and stronger immunosuppressive treatment is needed to tolerate the transplantation 5. In addition to this, another unavoidable complication of transplantation is ischemia-reperfusion injury (IRI). Its pathology also involves hypoxic injury during ischemia and oxidative stress and inflammation that occurs during reperfusion that results in the development of graft dysfunction, delayed recovery, and transplant failure 6. Also, technical barriers exist in the monitoring of the postoperative abode things are very few effective noninvasive ways of detecting the delayed graft function at an early stage and in managing the complications caused by IRI or immunosuppression 7. Novel approaches, including xenotransplantation, 3D bioprinting, nanoscale targeted delivery and diagnostics and mechanical perfusion, have been established to address the lack of organs, provide resistance against rejection, better graft preservation, and finally improve the graft function and long-term survival.

Nanomedicine is one of such strategies that has been extensively pursued in organ transplantation because of its size, which can be adjusted, surface properties customization, and targeting capability. It has carried out protective, regulatory and monitoring functions in the significant transplantation stages. Nanoparticles may be administered to eliminate and manage IRI by providing armed categories of active agents to curtail the circulation of pro-inflammatory cytokines and to control oxidative tension, effectively decreasing tissue pathophysiology 8. To resolve the problem of rejection, nanomedicine is capable of providing immunosuppressive agent at the site of grafting. This is the local method that would reduce side effects that may occur throughout the entire system, changes antigen-presenting cells to suppress immune response and fosters tolerance reducing the risk of rejection 9. Moreover, cellular and molecular alterations of the transplanted organ can be accurately followed with the help of nanotechnology-based image techniques. Such methods provide noninvasive postoperative diagnosis, which is effective and assists it to detect the graft dysfunction in its initial stages 10. Nanomedicine has therefore become a prospective transplantation medicine to aid in addressing the current clinical complications and enhance availability of organs and success of transplantation.

In this review, the mechanisms, designs, and applications of nanomedicine in organ transplantation are fully discussed. The main problems of organ transplantation have been described: invasive nature of biopsy-based checking of the organs, the inevitability of the effects of IRI at preservation, technical challenges of vitrification, and side effects of the existing immunosuppressants in dealings with the rejection. To solve these issues, benefits and designing concepts of nanomedicine are expounded. The strategy of nanocarriers to nanomedicine is classified and its targeting and ability to respond to stimuli are addressed. The use of nanomedicine in organ transplantation in recent applications is explained. Lastly, challenges of nanomedicine during translation into clinical practice are discussed and recommendations given on future directions. This review will offer a unified guide of application of nanomedicine in organ transplantation to enhance the living standards of transplant patients.

## 2. Current status and challenges in organ transplantation

**Figure 1:**
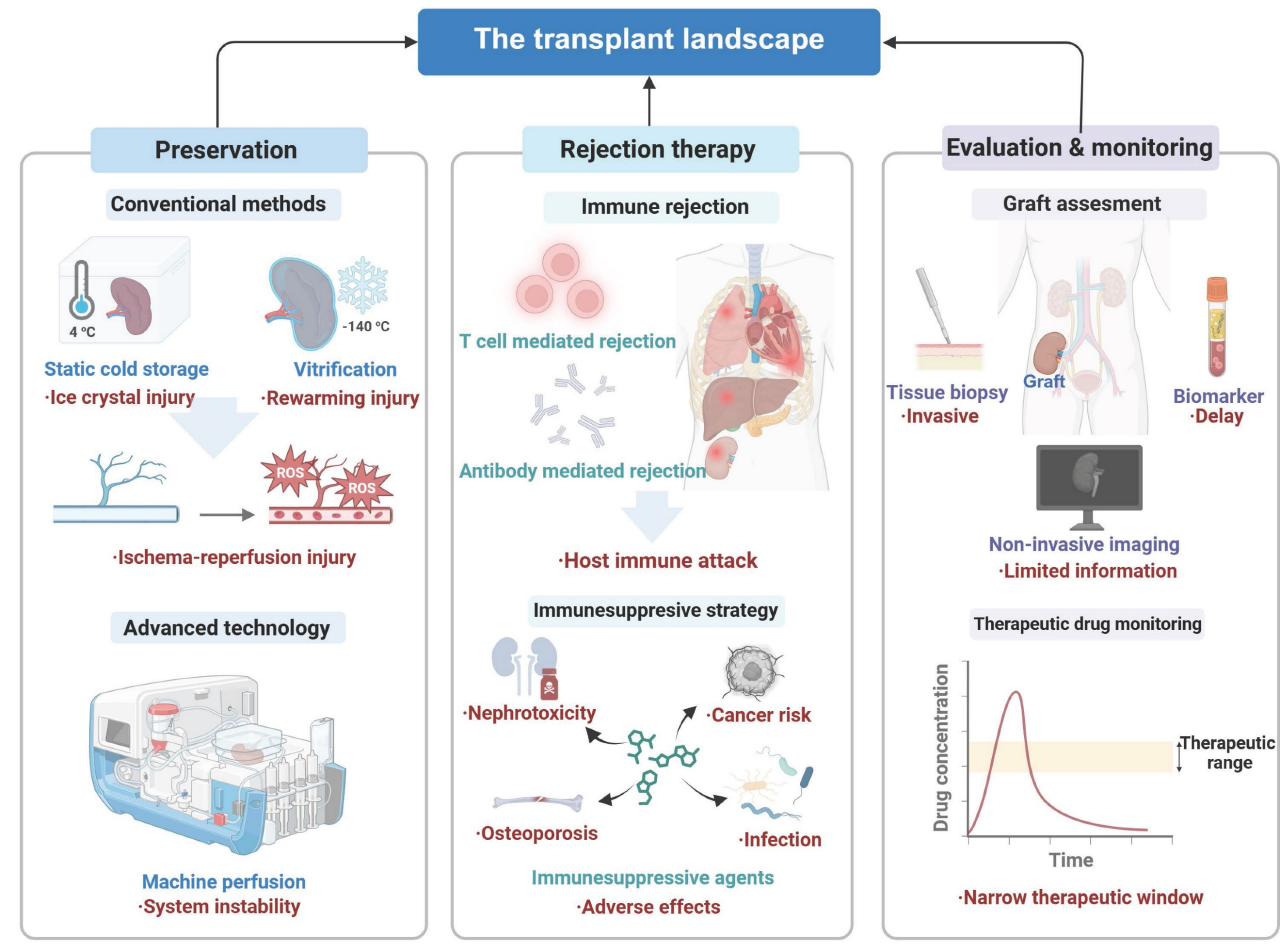
** Landscape of organ transplant management.** The standard clinical management of organ transplants involves (1) preservation methods such as static cold storage, vitrification, and machine perfusion, (2) rejection therapy by addressing immune rejection with optimized immunosuppressive strategies, and (3) comprehensive evaluation including graft assessment and therapeutic drug monitoring. Created with BioRender.com.

The long-term survival rate and the quality of life of patients after organ transplantation are modulated by a multitude of factors, encompassing current diagnostic technologies, the quality of preserved organs, rewarming techniques, IRI, and postoperative management of rejection. Currently, principal impediments for successful organ transplantation include invasive biopsy-based methods for organ evaluation, cold IRI during preservation, bottlenecks in cryopreservation and rewarming technologies, and the complexity of postoperative immune rejection in conjunction with very few immunosuppressive therapeutic options (**Figure 1**).

### 2.1 Organ preservation and IRI

Organ preservation is a critical stage whereby the physiological process and structural integrity of the graft is maintained after it has been taken off the body of the donor. This is a preservation process which helps in transporting organs and preparing them to undergo transplantation surgery and perform pre-transplant evaluations of organ viability and execution of organ repair measures. The inherent drawbacks of the preservation methods and unavoidable damage of transplants are some of the current issues faced in the preservation process 11. Preferably, organ preservation intervention would preserve the structural and functional integrity of the organ in an ex vivo state and enable the most protractable length of the transplantable period 12. Normal processes like static cold storage (SCS) result in the development of ice crystals to destroy intracellular and extracellular materials 13. In comparison, ultralow-temperature (-140 ^o^C) vitrification, involving CPAs and propylene glycol, freezes organs much faster to avoid the formation of ice and reduce the effects of ice on tissues, and allows the storage of organs over a long period. Although this is an advantage, the method is hindered by low success in thawing organs 14. Vitrified organs require rapid and constant warming to thaw successfully. Recrystallization is triggered by slow warming at between -40 °C and -60 °C, and the resulting ice crystals infiltrate and damage the cellular and mitochondrial membrane. The injury causes the discharge of damage-related molecular patterns (DAMPs) among other cellular content, which worsens oxidative stress and inflammation in case of severe IRI 15. Moreover, extensive organs experience internal stress due to imbalanced thermal conductive effects during thawing and may lead to structural damage. Although convective techniques are fast and evenly distributed and have been successfully used to warm small samples very fast, they are not yet prepared to scale to organ scale 16.

Machine perfusion (MP) as an organ preservation method and dynamic lie on its lab-bench discovery finding to clinical practice. MP has also solved the limitations of the conventional immobile cold storage by replicating physiological conditions to provide continuous access to nutrients and oxygen to improve the quality of organs and broaden the donor population 17. Two clinical modalities are commonly utilized, namely: NMP (normothermic machine perfusion) (35-38 °C) and hypothermic MP (0-12 °C). NMP is known to enhance metabolic activity and precise redox balance regulation is aware but hypothermic MP has the potential to compromise functional recovery by inhibiting metabolism 18. MP helps in the use of marginal donor organs by increasing the preservation period. It has been shown to transport and evaluate organs, support early graft functional repair, and deliver drugs to specific areas of the body to reduce systemic side effects 19. Nonetheless, MP has been faced with practical problems, whereby, the lengthening of the preservation time has been inadequate; functional recovery has been inadequate, and more organ loss has been witnessed as a result of system instability or perfusion failure 20.

An essential issue of MP to use as a mitigation strategy is IRI. The injury is started in the cold ischemia period and the duration of organ maintenance in a cold solution has a positive correlation with the amount of organ risks 21. Transplantation normally goes hand in hand with IRI. Reproximal restoration of a hypoxic tissue causes a major generation of ROS, which induces cellular injury and inflammation, which causes IRI 22. The impact of IRI varies significantly for different transplant types. In kidney transplantation, IRI causes acute tubular necrosis, delayed graft function, and reduced long-term survival 23; in liver transplantation, it often results in graft dysfunction and post-surgical mortality, with a continued lack of effective interventions 24; and in lung transplantation, it predominantly induces damage to an alveolar barrier and vascular endothelial cells 25. Additionally, IRI also reduces the quality of donor organs of extended criteria donors (ECDs) as they are prone to damage by virtue of an advanced donor age, comorbidity, or unstable capillary functions of the body. The organs are frequently wasted following IRI, a fact that enhances the global organ shortage 26. The existing clinical care of the IRI is not effective as a supportive care, and nanomedicine solutions have been researched in the context of amelioration of better targeting and regulated drug delivery.

Many donor organs are underused due to quality issues such as functional impairment or damage, as well as a higher risk of IRI. Tackling IRI during transplantation and repairing marginal organs could be innovative approaches to addressing these quality issues, and NMP perfusion emerges as a key platform for mitigating IRI through physiological maintenance and direct restorative effects 27. For instance, NMP helps reduce fatty degeneration to remove excess lipids in steatotic livers which are common from marginal donations 28. Moreover, grafts can be maintained in a perfusion system for several days before surgery, facilitating the clearance of potential infections 29.

### 2.2 Posttransplant anti-rejection therapy

**Figure 2:**
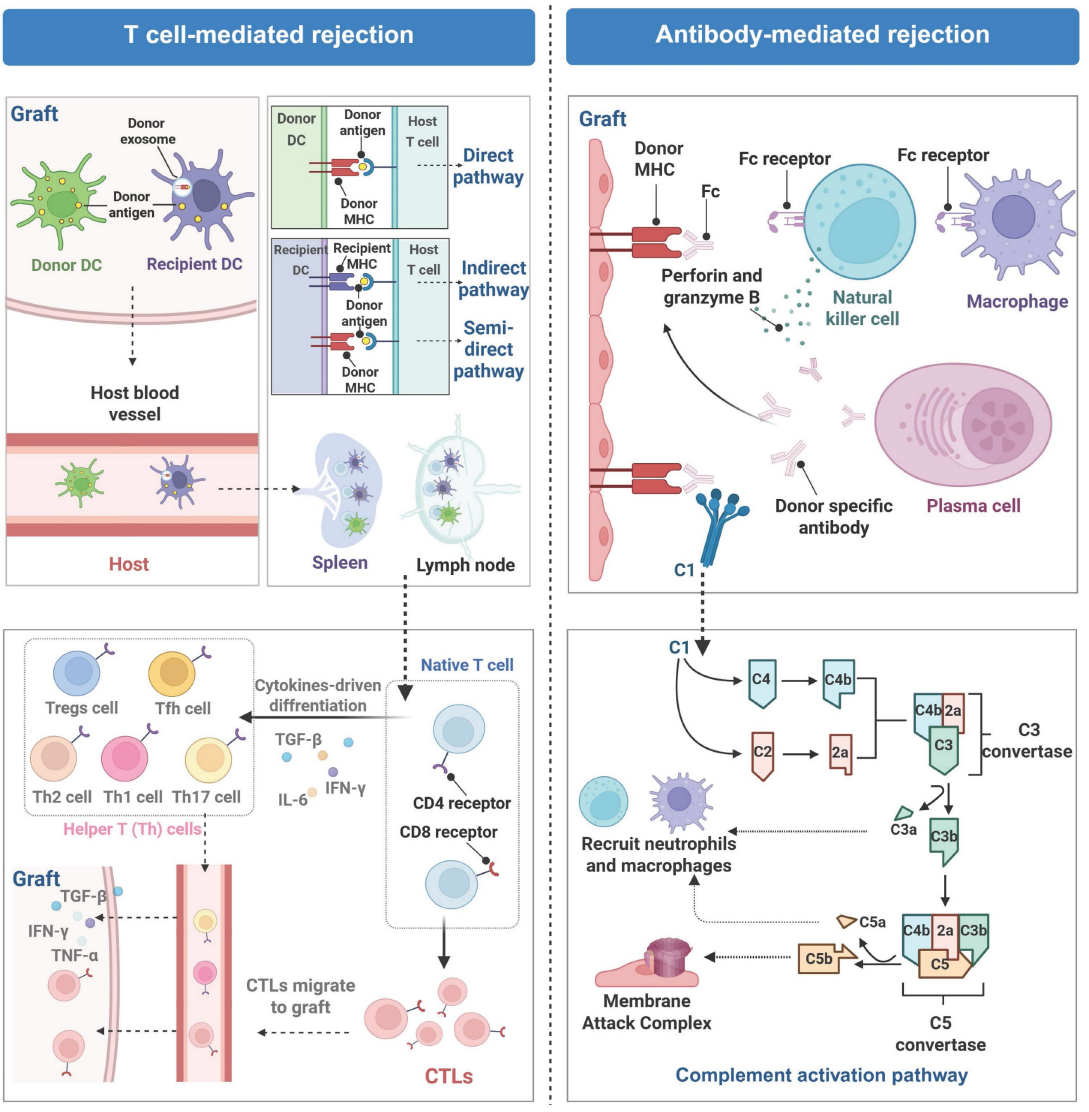
**Molecular mechanisms of T-cell-mediated rejection (TCMR) and antibody-mediated rejection (ABMR).** TCMR involves migration of dendritic cells (DCs) to lymph nodes and spleens, where they activate T cells to initiate cellular immune responses against the graft. ABMR is primarily driven by donor-specific antibodies, leading to Fc receptor-mediated engagement and complement pathway activation, which collectively lead to graft endothelial injury. Created with BioRender.com.

Rejection is a principal complication after organ transplantation and is characterized by an immune response triggered by the immune system of the recipient because the immune system treats the graft as a foreign or allogeneic tissue and initiates to eliminate it 30. This response is fundamentally a defense mechanism of the host against allogeneic antigens while it results in structural damage to the graft and loss of its biological function 31. On the basis of immune mechanisms and pathological characteristics, rejection is categorized into two primary types: T cell-mediated rejection (TCMR) and antibody-mediated rejection (ABMR). These two types may occur independently or coexist within the same graft (**Figure 2**).

TCMR is predominantly mediated by host effector T cells (including CD4⁺ and CD8⁺ T cells), and they initiate rejection responses by recognizing mismatched signals from human leukocyte antigens (HLA) between a graft from a donor and its original organ in the recipient via their T-cell receptors (TCRs) 32. Dendritic cells (DCs), professional APCs, present donor antigens through three pathways. The direct pathway involves recognition of foreign major histocompatibility complex (MHC)-antigen complexes on donor APCs by T cells; the indirect pathway involves recognition of donor antigens presented on their own MHC by T cells after processing by APCs. A recently discovered semidirect pathway involves acquisition of intact donor MHC-antigen complexes by recipient DCs through engulfing donor-derived extracellular vesicles (e.g., exosomes) and directly presenting them to naive T cells for antigen recognition 33. Upon recognition of antigens presented by APCs and costimulatory molecules, T lymphocytes become activated, undergo differentiation, and clonally proliferate. The activation results in the secretion of inflammatory cytokines to facilitate local immune cell infiltration at the site of the graft, leading to tissue damage via cytotoxic mechanisms and inflammatory responses 34. ABMR, which is characterized primarily by the presence of donor-specific antibodies (DSAs). DSAs specifically attack HLA class I or II molecules present on the surface of vascular endothelial cells of the graft and the attack is notably prevalent in cases of late-stage graft injury 35. The endothelial damage is realized through complement activation and Fc receptor-mediated effector pathways, which subsequently trigger the recruitment of inflammatory cells, including macrophages, neutrophils, T cells, and B cells, to infiltrate the graft 36. Acute ABMR typically manifests as tissue edema and vascular inflammation, whereas chronic ABMR results in intimal fibrosis 37.

In clinical practice, immune rejection is primarily managed through immunosuppression. The widely used drugs include tacrolimus (FK506) that blocks calcineurin; antimetabolites (e.g., rapamycin (RAPA) and everolimus) that curb immune cell growth; and glucocorticoids (e.g., methylprednisolone) that suppress inflammation and immune activity. Additionally, lymphocyte-depleting agents are used, including alemtuzumab, an anti-CD52 antibody, and rituximab, a B-cell-targeting drug 38-42. Although these treatments significantly improve short-term graft survival, long-term use of immunosuppressants carries severe risks, including a high rate of infection, cancer initiation, and chronic rejection 43. Furthermore, these agents often have a narrow therapeutic window and display off-target toxicity, which prevent their application over an extended period.

### 2.3 Organ evaluation and outcome monitoring

The evaluation of the overall life cycle of handling organ transplantation such as organ functioning, timely disease identification, and examining the effectiveness of treatment also allow dynamically assessing the gland functioning ability and assist in timely clinical decision-making. Nonetheless, transplantation experiences significant diagnostic and follow-up problems. To avoid use of substandard grafts, tissue biopsy, which is the gold standard way of detecting abnormality in donor organs, may be invasive and is associated with sampling errors, complications, high cost, and damage of organs 44. Moreover, noninvasive techniques that monitor tissue biopsy tend to fail to offer real-time but effective functional data of the donor organs, whether as a pre-transplant measure or even as a monitor to the organ recipient after transplantation. The tools that currently are noninvasive like the identification of blood or urine biomarkers are usually not specific or sensitive, which results in diagnosis delays 45, 46. The state-of-the-art imaging procedures like MRI cannot see through molecule level graft operation, thus hampering accurate treatments in transplantation medicine 47.

The main challenges in the real-time monitoring are presented by two factors in the sphere of real-time monitoring: monitoring dynamic concentrations of drugs and their therapeutic effects, detecting the dynamics of pathological processes, and their progress. Indicatively, FK506 that has been largely used in clinical practice has a remarkably small therapeutic index. Even minor changes in drug levels in blood may lead to the development of serious side effects, such as nephrotoxicity, or insufficient immunosuppression, so, very specific monitoring of the drug concentration in blood is the key of immunosuppressive therapy 48. Dynamic and complicated nature of IRI is what made the real-time monitoring of the pathological process hard. IRI plays an important role in dysfunction of posttransplant. There is a high speed of pathological processes, including localized reactive oxygen species (ROS) burst, mitochondrial dysfunction, and microenvironmental instability, hence, single-marker detection techniques cannot fully show the entire scope of damage 49. The conventional biomarkers of serum like transaminases and tissue biopsies are mainly used to ascertain the end-stage of tissue damage. Currently, there are no successful ways of detecting early signs and tracing dynamic development of IRI. The difference in type and quality of organ donor also is an added complication to diagnosis 50. Anxious oxidative injury patterns are revealed in different organs in IRI, and modern diagnostic tools are not able to recognize the differences between these patterns posing the threat of misdiagnosis. For instance, marginal donor organs, such as those from donors with fatty livers or elderly donors, are more vulnerable to IRI than organs from healthy donors. In the current diagnostic framework, these organ-specific physiological differences have not been comprehensively considered, and there is no standardized risk assessment to address the variation in the transplant 51. Therefore, highly sensitive diagnostic tools are needed to real-time monitor dynamic processes of IRI across different organ types and donor qualities. The mechanisms of rejection include TCMR, as well as ABMR or a combination of both. As each type involves distinct immune activation pathways, accurate molecular-level diagnosis requires combining multiple specific biomarkers.

## 3. Design strategies and principles of nanomedicine in organ transplantation

Nanotechnology has been harnessed to address key challenges in organ transplantation. It improves diagnostic precision by enhancing target specificity and amplifying signals in a diseased area, and it allows the integration of high sensitivity, noninvasiveness, and multimodality in a single platform for earlier and more accurate real-time diagnosis. Meanwhile, nanomedicine has been used for organ preservation through magnetically induced uniform rewarming and targeted delivery of protective agents to reduce oxidative stress and inflammation caused by IRI. These nanomedicine approaches help improve the organ quality and expand the donor pool. Moreover, advanced nanocarriers have been innovatively applied to optimize immunosuppressive therapy by enhancing solubility and bioavailability of poorly soluble drugs including FK506 and RAPA. Through targeted design, nanocarriers help achieve localized drug accumulation at the graft site, effectively reducing systemic drug exposure and improving drug safety.

### 3.1 Fundamental design principles for nanomedicine in organ transplantation

Nanomedicine is a drug delivery system in which active pharmaceutical ingredients are encapsulated within nanoscale carriers. Drugs are loaded onto these nanocarrier systems through encapsulation, adsorption, or chemical bonding to realize targeted drug delivery and responsive activation to specific stimuli 52. Compared with traditional pharmaceutical formulations, a high specific surface area of nanomedicine enhances the bioavailability of a barely soluble drug and protects the active component from premature degradation. They can be actively or passively deposited in diseased tissues or in certain cells through functional alterations and, therefore, toxic by side effects on healthy tissues are reduced as much as possible 53. Moreover, nanomedicine was also used in the noninvasive diagnostics to evaluate pathological conditions and therapy responses in real time 54. Thus, nanomedicine undoubtedly has opportunities to improve the results of treatment and the quality of diagnostics, and the opportunities in the field of organ transplantation can be exploited to overcome the obstacles. Accurate and effective nanomedicine specific to organ transplantation can be designed through the employment of fundamental principles of design of nanocarriers.

**Figure 3:**
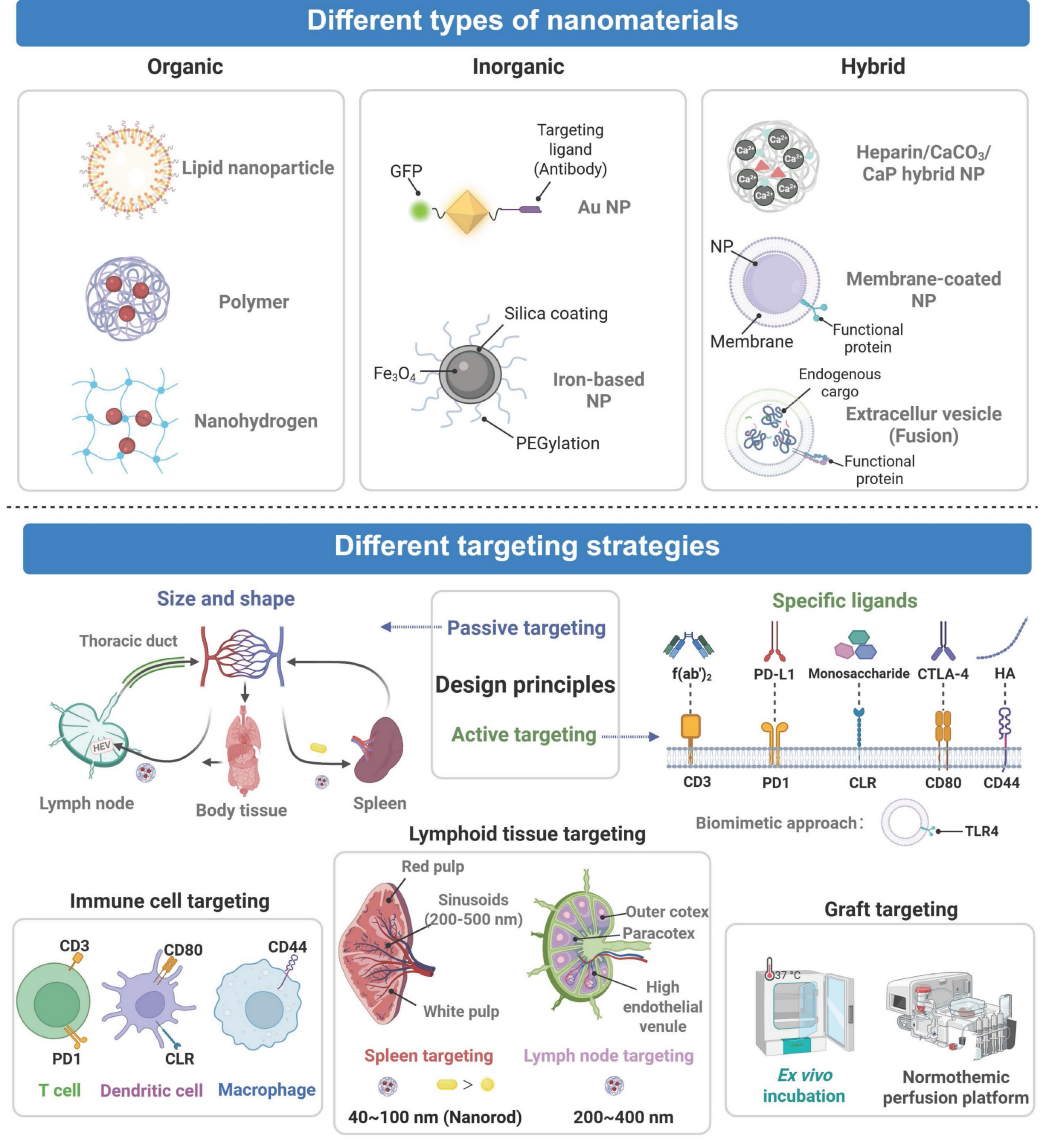
** A framework for nanomedicine in transplantation.** Nanomedicine in transplantation employs engineered nanomaterials (organic, inorganic, hybrid) and rational design to achieve precise targeting of lymphoid tissues, immune cells, and grafts by employing either active or passive strategies. Created with BioRender.com.

The main design concept of the nanocarrier is to adjust physicochemical characteristics of its resulting nanomedicine to provide targeting delivery and homogenous distribution in the transplanted organs, such as size, surface charge, and functionality moiety alterations 55. These properties are optimally useful in minimizing systemic exposures of the immunosuppressants to normal tissues. The size of nanomedicine is a very important factor in transplantation site as it travels through the biological barriers. For example, due to difference in microcapillary endothelial gaps in distinct organs, kidney (70-90 nm), liver (50-100 nm), and spleen (200 nm), nanomedicine that has been prepared at a given size range will easily traverse the respective capillary walls, thus increasing organ-specific accumulation 56. It has been established that nanomedicine with very small size (less than 5mm) is rapidly excreted through the kidneys, but the large size (more than 200mm) of nanomedicine is readily taken up by the mononuclear phagocyte system (MPS). Thus, the optimization of off-target effects requires the optimization of the particle size 57. Also, nanomedicine has an effect on delivery based on its surface charge. Nonspecifically binding of positively charged nanomedicine to cell membranes and neutral or slightly negative biosensing of nanomedicine surfaces have been observed to minimize protein adsorption and increase the circulation 58. Additionally, the nanomedicine can be modified to include functional moieties that can help in advancing the precision of the immunosuppressant delivery. Indicatively, nanomedicine modification with polyethylene glycol (PEG) renders high effectiveness in extending the systemic circulation period 59. Specific targeting ligands conjugation confers upon nanomedicine the capacity of accurately identifying and accumulating in particular organs or cell kinds thus enabling accurate immune modulation 60 (**Figure 3**).

Additionally, stimuli-responsive nanomedicine is highly beneficial in that it can react to endogenous or exogenous signals, and with specificity of the drug release process via regulated physicochemical changes. Diagnostic functions are combined with therapeutic functions in nanomedicine, which allows constant monitoring and regulation of the therapeutic intervention during the organ transplantation process 61. As a summary, these general principles can help develop nanomedical designs useful in organ transplantation to reach the targeted, controlled drug delivery, integrated diagnosis and treatment. This approach synergistically enhances the efficacy of immunosuppression, protects transplanted organs from IRI, supports noninvasive monitoring and realizes personalized precision medicine after transplantation (**Table 1**).

### 3.2 Classification of nanocarriers for organ transplantation

Nanomedicine used in organ transplantation can be classified into organic, inorganic, and hybrid nanomaterials according to their material composition and each category has distinct structural and therapeutic advantages. Organic nanomaterials, including polymeric nanoparticles and lipid nanoparticles (LNPs), are highly biocompatible and biodegradable. Their surfaces can be readily functionalized with targeting molecules to achieve precise drug delivery 92. The most representative polymer, PLGA, exhibits excellent biocompatibility and low immunogenicity. PLGA has been used to effectively encapsulate immunosuppressants and realize sustained drug release. For instance, Deng *et al.* developed FK506-loaded PLGA nanoparticles that helped prolong the survival of cardiac grafts in a rat model 62. In addition, LNPs are composed of biocompatible lipids and their physicochemical properties can be precisely regulated. They have been employed to efficiently encapsulate and deliver nucleic acids, proteins, and small-molecule drugs to achieve potent targeted delivery. It has been recently reported that liver-targeting LNPs composed of an ionizable lipid SM102, DSPC, and cholesterol were applied to encapsulate SIRT4 mRNA via microfluidic technology. These LNPs increased the level of SIRT4, hindered ferroptosis, and decreased IRI following the liver transplantation 63. PEG and polyacrylamide are organic polymeric coatings that enhance biocompatibility and extend systemic circulation and increase the therapeutic activity 93, 94. Indicatively, PEG-ami-PEGylation was incorporated into an oligolycine surface to lower the non-specific uptake of nanoparticles by liver endothelial cells, which reduce clearance by the liver and enhance its targeting 64. Natural polysaccharides are also good in the construction of nanomedicine since they are not toxic and immunogenic. The biomaterials include chitosan, hyaluronic acid, trehalose, dextran and heparin which are very cheap but are highly biocompatible naturally. They contain polysaccharides that facilitate the uptake into cells and allows the long release of the drug to be performed 95. Nanohydrogels based on polysaccharides contain a three-dimensional crosslinked structure that has high water absorption and retention. They are composed of a similar material like one of a natural tissue and their application can result in a lower chance of immune rejection post-transplantation 96. As an example, a porous chitosan amino acid hydrogel has been reported to be able to create a good sustenance of drugs release. It is a biodegradable and biocompatible hydrogel that can be used in delivery of drugs locally 65.

The inorganic nanomaterials have unique benefits in transplantation of the organ as they are structurally rigid, stable and tunable 97. Their pores and surfaces can be decorated to form artificial nanosystems that mimic an antioxidant nanoenzyme or can be used to carry a drug to modulate the immune system. As an example, Feng *et al.* designed gold platinum nanoparticles (AuPt NPs), which had a catalase-like activity and efficiently eliminated ROS to alleviate renal IRI during the transplantation procedure without any noticeable toxicity 66. Gold nanoparticles are known for their low toxicity and biocompatibility. Their intrinsic safety can be enhanced through organic functionalization, and this functionalization process can also improve stability and biocompatibility of gold nanoparticles. Surface modification of gold nanoparticles with targeting ligands enables localized drug accumulation in transplant organs, supporting targeted therapy 98. In a recent study, gold nanoparticles conjugated with an anti MHC class II antibody were selectively bound to MHC class II positive cells. Labelling of these particles with radioisotopes allowed PET/CT imaging, thus this platform could be used for post-transplant immune monitoring and targeted treatment 68. Iron-incorporated nanomedicine, which has an iron-containing core, can be directed to transplanted organs under a magnetic field to reduce systemic toxicity of drugs in nanomedicine. They also act as an MRI contrast agent to allow real-time, noninvasive organ monitoring 99. Additionally, iron-incorporated nanomedicine exhibits a magnetothermal property under an alternating magnetic field, and it can be harnessed for uniform rewarming of organs 100. These properties support that the inorganic nanomedicine can be multifunctional to improve graft survival after combination of diagnosis and treatment.

Hybrid nanomaterials integrate diverse inorganic and organic components, such as metals, polymers, or biomolecules, to create nanoscale systems with novel composite structures. This approach overcomes the limitations of pure inorganic or organic systems, enabling enhanced functionality and broad application prospects 101. An example is calcium phosphate (CaP), calcium carbonate (CaCO_3_) which has a high drug loading capacity. CaCO_3_ is degraded in a responsive manner in an acidic environment and controlled release of drugs can be done once CaCO_3_ has degraded 102. The addition of heparin prevents aggregation of inorganic nanoparticles through steric hindrance and electrostatic repulsion and enhances their colloidal stability. Therefore, the heparin/CaCO₃/CaP hybrid nanoparticles could be used to build a stable drug delivery platform 69. Another example is a biomimetic platform (PEI arg@MON@BA) prepared from degradable silica. Four functions including cfDNA adsorption, ROS clearance, calcium chelation, and NO release were integrated in this platform to significantly reduce IRI in rat and human liver samples 67. Furthermore, by incorporating unique functions from natural biological components, hybrid nanomaterials demonstrate significant advantages in the field of organ transplantation, including low immunogenicity, excellent biocompatibility, and active targeting capabilities. Two representative biomimetic nanomaterials are cell membrane-coated nanoparticles and extracellular vesicles. Cell membrane-coated nanoparticles retain the properties of the membranes of their source cells. For example, a protective effect of macrophage membrane-coated nanoparticles (M-NPs) against liver IRI was demonstrated in a rat orthotopic liver transplantation model 70. Toll-like receptor 4 (TLR4) from macrophage membranes was preserved on these M-NPs, and it specifically neutralized the endotoxin LPS and suppressed LPS-induced macrophage activation. The macrophage membrane coating also helped migration of the nanoparticles into inflamed areas while improving biocompatibility and immune evasion. Innovative multifunctional fusion extracellular vesicles (FNVs@RAPA) were developed to treat early IRI and immune rejection after heart transplantation 71. These vesicles were created by fusing nanovesicles (ENVs) from Aurantium chinensis with mesenchymal stem cell membrane-derived nanovesicles (CNVs) with overexpressed calreticulin (CALR), and the formed functional carriers were used for RAPA delivery. Plant-derived ENVs displayed low immunogenicity in animal models. They contained a myriad of miRNAs and anti-inflammatory molecules that could provide antioxidant and anti-inflammatory effects during the organ transplantation process. Meanwhile, CNVs retained the immunomodulatory function and they could target macrophages.

Although few nanomedicines are currently clinically used in organ transplantation, the nanocarriers for nanomedicine have advantages of diverse types, functional integration, and favorable biosafety, and the resulting nanomedicine could be clinically applied for targeted therapy for transplanted organs, immune microenvironment regulation, and injury repair.

### 3.3 Targeting strategies of nanomedicine for organ transplantation

Traditional organ transplant therapies suffer from toxic side effects of systemic immunosuppression. Targeted nanomedicine delivery can improve the precision of immunosuppressant delivery, thus enhancing both safety and efficacy. The spleen and lymph nodes play central roles in transplant immunology. The spleen, the largest lymphoid organ, contains diverse immune cells that regulate systemic immunity. Lymph nodes function as hubs for adaptive immunity and coordinate T cell activation and immune surveillance. The spleen and lymph nodes are critical in both rejection and tolerance after transplantation. Consequently, targeted delivery of immunomodulators precisely to the spleen and lymph nodes can reduce their side effects and enhance their therapeutic efficacy. Nanomedicine possesses two categories of principles of targeting, including passive and active targeting. Passive targeting is contingent on the size and surface characteristics of the nanocarriers and natural localization of nanomedicine into organs or locations might be attained by passive targeting. As an example, the MPS identifies some nanoparticles in its natural state, and it may be concentrated in the immune organs, e.g. the thymus and lymph nodes 103. Active targeting, in its turn, would entail altering nanomedicine in the form of targeting ligands. These ligands have the ability to personally identify receptors on involved organs or cells and bind, and this significantly improves the delivery accuracy and efficacy 104.

The stromal cells, the medullary cells, and the lymphocytes are found in lymph node (LNs) which are secondary immune organs 105. They possess connective tissue capsule and comprise of cortex as well as medulla. The outer cortex contains the B cells and the inner paracortex is where T cells, DCs, and high endothelial venules (HEVs) which give access to lymphocytes via blood vessels. B cells, plasma cells and macrophages are found in the cords in the medulla and sinuses are filtered by the medulla. Lymph goes in the conduits and sine to facilitate contact between the antigens and the immune cells. On reaching the local LNs through lymph, transplant derived antigens stimulate allogeneic T cells thereby causing an immune response against the graft 106. Nanomedicine is capable of being used to specific LN structures and cells and this mode has come to be one of the major strategies of reducing rejection. Nanomedicine is generated by targeting lymph nodes, and the size of the particles plays a major role in the delivery process. McCright *et al.* reported the successful accumulation of PEG-modified nanoparticles (40 to 100nm) in lymph nodes four hours following intradermal injection, which was due to their increased hydrophilicity and lower clearance. Particles of high-density PEG coating (40 nm) showed the greatest rate of accumulation in lymph nodes amongst them 72. The specificity of nanomedicine is further enhanced following the incorporation of special ligands of targeting. Qiu *et al.* constructed the tannic acid-conjugated nanocarrier (TA-FNP) which was able to interact with the elastin of endothelium lymphatic. This carrier is a carrier that has FK506 and it is loaded in the carrier at a size of around 86 nm that is used to deliver FK506 to the lymph node. TA-FNPs infiltrated lymphatic endothelial junctions in the paracortical region to release FK506 to suppress T-cells activation and multiplication. In a heart transplant model, this system significantly reduced T-cell infiltration into grafts and prolonged graft survival 73.

The spleen is the largest peripheral immune organ in the body and contributes to immune responses and blood filtration. Its parenchyma consists of white pulp, red pulp, and a marginal zone between them 107. The white pulp, organized around the central artery, is the main site for lymphocyte accumulation and immune activity. It contains the periarterial lymphatic sheath predominantly populated with T cells and lymphoid follicles enriched with B cells. The majority of the splenic parenchyma is composed of the red pulp which is constituted with a splenic cord and sinusoids. The splenic cord is a reticular structure containing rich macrophages, plasma cells, and blood cells, and it plays an important role in removing old red blood cells and capturing foreign particles from the blood. Splenic sinuses are specialized venous structures with gaps of about 200-500 nm wide, allowing slow passage of blood cells and capture of macrophage-mediated particles. The marginal zone has a clear organisation having numerous macrophage DCs and B cells. In the area, lymphocytes are activated by antigens in blood that is carried in the blood 108.

The spleen plays a role in graft damage during transplant rejection through antigen capture, T cells activation and antibody production of the donor antigens. The spleen has become a vital object of nanomedicine because of its primary location in immunity. Nanocarriers can be designed for splenic accumulation through precise control of the particle size and tuning of surface properties. For example, nanoparticles at a size of larger than 200 nm or those with hydrophilic coatings have exhibited enhanced splenic retention 109. However, uptake of these nanoparticles by hepatic Kupffer cells has frequently diminished the effectiveness in their splenic delivery. To address this impediment, approaches including hydrophilic coatings, biomimetic modifications, and non-spherical designs have been developed to help reduce liver clearance and improve splenic targeting 107. Gu *et al.* compared the distribution of poly(*N*-isopropylacrylamide) (PNIPAM)-coated nanostructures with different morphologies in mice, including spheres, rods and rings 74. Non-spherical particles exceeding 400 nm, particularly rods and rings, accumulated preferentially in the red pulp, which could be ascribed to structural constraints of splenic sinusoids with 200-500 nm endothelial gaps. To apply this discovery in transplant immunosuppression, an FK506-loaded nanospiral composed of phosphatidylserine (PS70) and cholesterol was developed for targeting the spleen and lymph nodes 48. The anionic PS70 component promoted uptake by mononuclear phagocytes. This nanospiral helped elevate the FK506 level in both the spleen and lymph nodes. This nanomedicine displayed precise targeting, high efficacy, and great safety over conventional formulations and it could be promising as a novel immunomodulatory candidate for transplantation.

Besides acting on immune organs, nanomedicine also has the ability to regulate immune responses through the specific targeting of immune cell populations, e.g. T cells, DCs, and macrophages. This strategy enhances the effectiveness of transplant rejection treatment besides decreasing systemic toxicity 110. Considering that T cells are the key actresses of graft rejection, T cell-based active targeting interventions raise special interest. Through surface functionalization with antibodies or ligands against targets on T cells such as PD-1 or CD3, nanomedicine can overcome the nonphagocytic nature of T cells and enable efficient drug internalization and release 111. For instance, Kim *et al.* developed anti-CD3 antibody fragment-modified nanomedicine (aCD3/F/ANs) to enhance T cell targeting through CD3-specific binding and active internalization 75. It was confirmed that aCD3/F/ANs, via their surface antibodies, significantly enhanced T-cell-mediated active internalization by specifically binding to CD3, a key component of the TCR complex. A gellan gum-based nanogel (npGG) conjugated with anti-CD3 and anti-CD28 antibodies was developed. This system mimicked activation of natural T cell receptors, providing a novel strategy for intervention of transplant rejection 76.

DCs, principal APCs, regulate transplant rejection and immune tolerance by controlling T-cell activation. Nanomedicine can deliver immunomodulators to DCs through targeting molecules on the surface, such as specific sugar ligands or antibodies. This approach can promote DC maturation and enhance antigen presentation, thereby strengthening regulatory DC induction to suppress allogeneic immune responses 112. For example, AuNP modified with mannose, galactose and fucose at 30 different combination ratios to target C-type lectin receptors highly expressed on DCs. These monosaccharide-coated nanoparticles achieved precise delivery of immunosuppressive agents, and displayed significant potential in improving immune tolerance and reducing rejection 77. In addition, Xu *et al.* integrated genetic engineering with nanotechnology to construct dual-targeted nanovesicles to target T cells expressing both programmed death-ligand 1 (PD-L1) and cytotoxic T-lymphocyte-associated antigen 4 (CTLA-4). These nanovesicles simultaneously inhibited T-cell activation by PD-1/PD-L1 interaction, they blocked CD28 costimulatory signals by CTLA-4/CD80 interaction on DCs and suppressed DC antigen presentation leading to synergistic immune suppression 78.

Macrophages dominate IRI and rejection and have now become a fundamental focus of nanomedicine designs. Nanomedicine provides an opportunity to modulate the macrophage activity and thereby reduce cell inflammation, inhibit proinflammatory molecules, and improve the graft survival 113. Ligand modification can be done using nanocarriers with active targeting. An mTOR inhibitor, PP242, was conjugated to a prodrug nanoparticle (DPNP) by means of conjugation to the docosahexaenoic acid (DHA). DHA was effectively absorbed by the macrophages through lipid metabolism pathways hence promoting effective uptake of drugs and dramatically increasing the lifespan of cardiac grafts 114. In addition to ligand modification, biomimetic nanocarriers, including mesenchymal stem cell membrane-based ones, can be macrophage-specifically targeted using the natural membrane proteins coupled with enhanced drug delivery efficiency to injury sites 71. M2 macrophages upregulate receptors like CD44 and interaction between hyaluronic acid (HA) and CD44 triggering clathrin-mediated endocytosis and achieves specific delivery 80. By harnessing this mechanism, a HA-coated nanoparticle (HATM) was designed to actively target M2 macrophages and renal tubular epithelial cells in IRI-affected kidneys, and its accumulation was pronounced in inflamed tissues. Therefore, macrophage-targeting nanomedicine has shown therapeutic potential for renal IRI 71.

Nanomedicine can be engineered to deliver therapeutic agents precisely to transplanted organs to enhance organ protection and functional recovery. For instance, Yu *et al.* utilized LNPs at a size of approximately 100 nm to achieve passive hepatic accumulation through hepatic sinusoids with high permeability. These LNPs were endocytosed by hepatocytes to deliver encapsulated SIRT4 mRNA to mitigate hepatic IRI 63. It was shown that distinct hepatic accumulation at 24 hours after administration, with minimal distribution to the spleen and lungs, confirming effective liver targeting by LNPs. In another investigation, Pandolfi *et al.* developed a targeted nanomedicine, GNP-HClm, by loading imatinib onto AuNPs functionalized with anti-CD44 antibody fragments to treat bronchiolitis obliterans syndrome after lung transplantation 81. GNP-HClm was bound to CD44 glycoprotein highly expressed on fibroblasts to realize active targeting. This strategy resulted in an elevation in the drug level at lesion sites, and the released drug exerted significant antifibrotic and immunomodulatory effects *in vivo*.

In addition to *in vivo* targeted therapy, nanomedicine can be employed for precise intervention in donor organs during *ex vivo* perfusion. It has been documented that a dendritic polyethylene glycol (PEG-dendron) nanomaterial, which can adhere to a pancreatic islet surface to achieve localized immunosuppression after release of the immunosuppressive drug in the nanomaterials, thereby improving islet transplant survival in a diabetic model 82. When the nanomaterial was incubated with isolated islets, the NHS ester groups on PEG-dendron formed covalent bonds with amino groups on islet cell surface proteins to create a physical barrier that reduced immune recognition. Additionally, normothermic machine perfusion can be served as a delivery platform for localized nanotherapy. Tietjen *et al.* developed poly (lactic acid)-polyethylene glycol (PLA-PEG) nanoparticles functionalized with anti-CD31 antibodies, and the nanoparticles specifically bound to CD31 molecules on renal vascular endothelial cells 83. When administered during *ex vivo* perfusion, these nanoparticles accumulated effectively in renal endothelial cells, and efficient targeted therapy was achieved in donor organs.

### 3.4 Stimuli-responsive strategies for nanomedicine in organ transplantation

**Figure 4:**
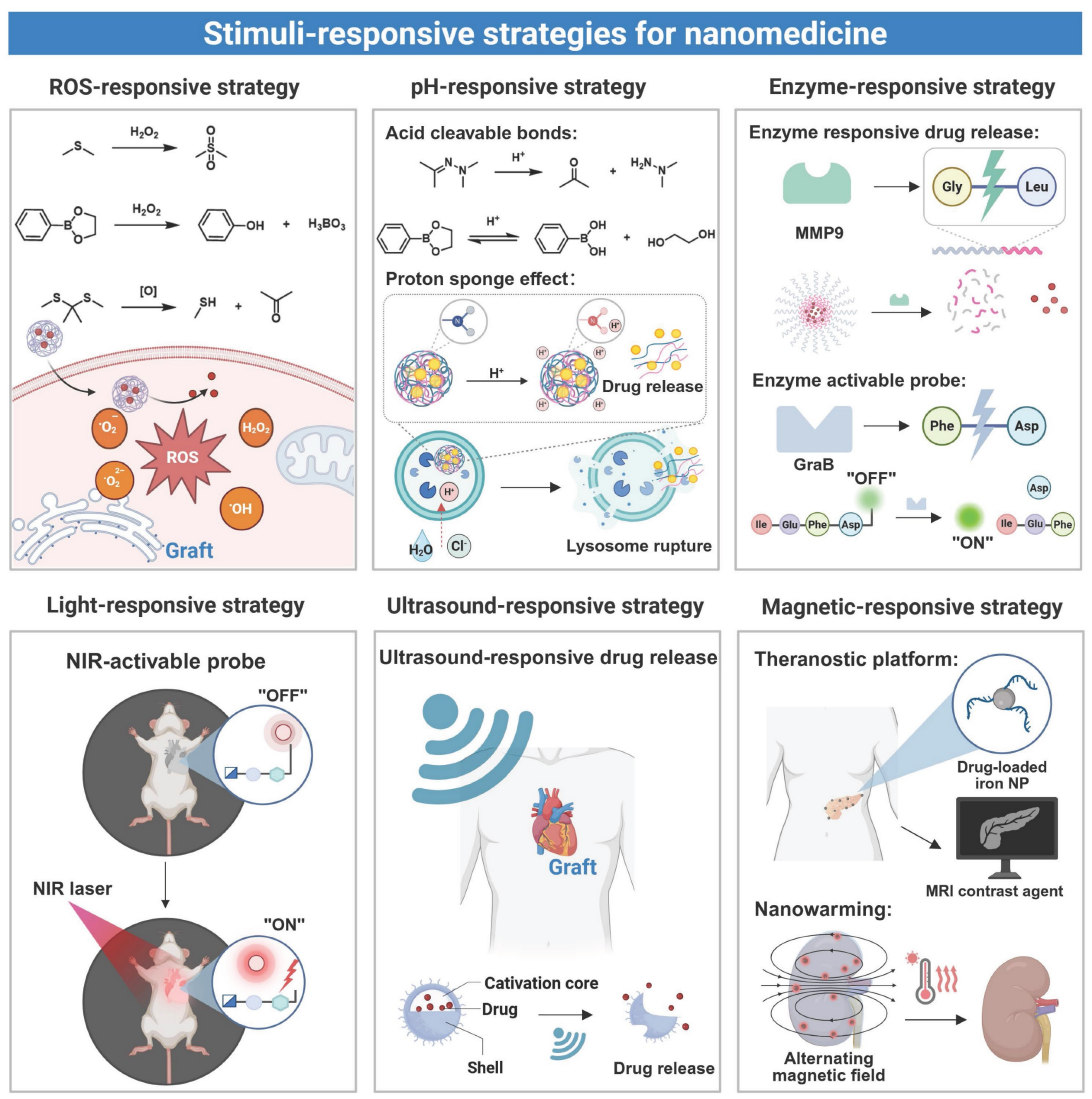
** Stimuli-responsive strategies for nanomedicine in transplantation.** Endogenous triggers (ROS, pH, enzymes) and exogenous stimulus (light, ultrasound, magnetic fields) are harnessed to enable controlled drug release and activate nanoprobes at targeted sites. Created with BioRender.com.

Endogenous stimuli-responsive design of nanomedicine is based upon biochemical changes within an inflammatory microenvironment of grafts, and the stimuli trigger drug release from the nanomedicine through mechanisms including bond cleavage or carrier transformation 115. Exogenous stimuli-responsive nanomedicine undergoes physicochemical changes upon exposure to external sources like light, a magnetic field, or ultrasound, allowing precise spatiotemporal control over drug delivery and activation. Both approaches have been demonstrated to improve therapeutic delivery precision and enhance diagnostic sensitivity/accuracy during transplantation 116 (**Figure 4**).

In organ transplantation, IRI or immune rejection often leads to reactive oxygen species accumulation, a decrease in pH, and an elevation in the expression level of certain enzymes. These endogenous signals have been acted as molecular triggers to stimulate nanomedicine to release loaded therapeutic agents at the lesion site. This localized delivery strategy reduces systemic drug exposure and minimizes off-target effects. The ROS-responsive nanomedicine has been developed to achieve targeted drug release in response to ROS at an abnormally elevated level in a pathological microenvironment. In this type of nanomedicine, specific chemical bonds, such as sulfur-containing or ester linkages, undergo cleavage upon interaction with ROS 117. ROS-responsive nanoplatforms for targeted drug delivery have been applied in transplantation. For example, a drug delivery platform (PMON@Pt) based on a mesoporous organosilica system (MON) was developed by incorporating tetrasulfide bonds and surface-functionalized phenylboronic acid pinacol ester. In a high-ROS microenvironment of liver IRI, this nanoplatform underwent degradation through boronic ester cleavage and matrix disruption, achieving efficient release of platinum nanoparticles 84. In another study, a chitosan-based nanoplatform modified with phenylboronic ester was developed for the delivery of myricetin 118. The phenylboronic ester acted as a ROS-responsive linkage, which underwent specific oxidative cleavage after exposure to a high level of H₂O₂ present in hepatic IRI, thereby triggering rapid release of myricetin. The released drug effectively exerted antioxidant effects and promoted the repair of damaged vascular endothelium. Similar results were found in HATM micelles which employed thioketone (TK) linkages to achieve selective drug release at renal IRI sites 80. In addition, a ROS-responsive azide-glycoside precursor (ROS-N₃) connected via phenylboronic ester bonds was developed. Upon H₂O₂ exposure, an azide group that was anchored on cell membranes was exposed in the cleaved product, enabling subsequent bioorthogonal conjugation with dibenzocyclooctyne (DBCO)-modified nanovesicles for site-specific drug delivery after heart transplantation 71. These strategies collectively demonstrate the therapeutic potential of ROS-responsive nanomedicine during the transplantation process.

pH-responsive nanomedicine achieves targeted drug delivery by harnessing the pH difference between inflamed sites and their neighboring healthy tissues. Transplant rejection creates a localized acidic microenvironment, while normal tissues maintain a neutral pH, and the pH difference can be explored for selective drug release from nanomedicine 119, 120. pH-response mechanisms include protonation-induced charge changes, alterations in hydrophobic interaction, and cleavage of acid labile bonds 121. Nanocarriers maintain a neutral or negative surface charge during circulation, and their surface charge is changed to be positive in an acidic region, improving their binding to target sites. Don *et al.* developed a pH-responsive nanomedicine through self-assembly of chitosan and fucoidan to deliver curcumin 122. The carrier response mechanism was contingent on the protonation state of the amino groups of chitosan under a specific pH condition. In a physiologically neutral environment, the amino groups remained deprotonated, whereas the amino groups protonated and acquired a positive charge upon entering an acidic microenvironment of an inflamed site. The positive surface charge of the nanomedicine enhanced its interaction with negatively charged inflammatory cells, facilitating cell binding and endocytosis. In addition to the charge reversal mechanism, another prevalent approach to achieving pH-responsive release involves the utilization of acid-labile chemical bonds (e.g., ester, hydrazone, or hydrazine bonds). A recently developed hyaluronic acid-based pH-responsive nanoparticle (HRRAP NP) achieved codelivery of all-trans retinoic acid (ATR) and RAPA 85. All-trans ATR was conjugated to HA through a hydrazone bond, and the resulting conjugate self-assembled into nanoparticles. During the self-assembly process, RAPA was encapsulated in the hydrophobic core of nanoparticles. Under an acidic inflammatory condition, cleavage of the hydrazone bond triggered nanoparticle disassembly, realizing simultaneous drug release. These nanocarriers often contain weakly basic groups, such as imidazole or amino moieties, to strengthen the proton sponge effect. During lysosomal acidification, these weakly basic groups absorbed protons, and the osmotic pressure was increased to cause lysosomal rupture and facilitate cytoplasmic drug release 123. In another example, a pH-responsive lipid nanoparticle was designed for microRNA delivery 86. An ionizable lipid, DODAP, was employed in this nanoparticle, and it maintained neutrality at a physiological pH to minimize nonspecific clearance and extend the circulation time. Upon entering acidic lysosomes in cardiomyocytes, the tertiary amine group of DODAP underwent protonation, enhancing the endosomal escape efficiency and promoting cytoplasmic release of microRNA to treat post-transplant rejection.

Enzyme-responsive nanomedicine undergoes structural changes after exposure to overexpressed enzymes in the transplant microenvironment to achieve localized drug release 124. For instance, matrix metalloproteinase 9 (MMP9), upregulated during liver transplant rejection, accelerated degradation of extracellular matrix components 125. By harnessing this mechanism, Luo *et al.* developed an MMP9-responsive delivery system (PLG-g-LPEG/TAC) for targeted TAC release to treat acute liver rejection 87. When nanoparticles accumulated in the rejected liver tissue, upregulated MMP9 cleaved the Gly-Leu peptide bond to trigger micelle dissociation and rapid TAC release. This approach improved the liver function and survival in a rat model. Additionally, to target granzyme B (GraB) expressed in inflammatory lesions, a bioluminescent probe (GBLI-2) was designed by conjugating a specific substrate IEFD with D-fluorescein. Cleavage of the bond between IEFG and D-fluorescein by GraB resulted in release of D-fluorescein and generation of light signals. This approach allowed sensitive monitoring of the GraB activity for real-time detection of immune activation and rejection after transplantation 88.

Externally activated nanomedicine can be precisely controlled for drug release to improve both therapeutic efficacy and safety. Light is a commonly used external stimulus, and it can induce structural changes in nanocarriers through photocleavage or photoisomerization mechanisms 126. Photoresponsive nanocarriers are stimulated by two types of light at different wavelengths: short-wavelength light (UV-visible, 100-780 nm) and near-infrared light (NIR, 780-1400 nm). Short-wavelength light displays a short tissue penetration depth, triggers premature drug release and induces tissue damage 127, while NIR allows deep penetration with reduced phototoxicity, and it has been employed to target large transplanted organs, such as kidneys, livers, and hearts 128. The majority of current light-responsive nanomedicines are activated by short-wavelength light because chemical bonds in these nanomedicines show weak response to NIR. To address this issue, a NIR-activated dual-responsive nanoprobe (UCNP@mSiO₂@SP-NP-NAP) was designed for simultaneous detection of hydrogen polysulfide (H₂Sₙ) and sulfur dioxide (SO₂) in myocardial IRI 89. This dual-responsive design allowed dynamic, simultaneous monitoring of both SO₂ and H₂Sₙ, enabling precise assessment and real-time visualization of IRI in transplanted organs.

Ultrasound responsive nanomedicine utilizes the deep penetration and noninvasive properties of ultrasound for targeted drug delivery to transplanted organs 129. This is done through conversion of acoustic energy to localized pressure variations to cause microbubble oscillation and collapse, which cause very strong mechanical stresses and specific thermal effects on nanomedicine, which contributes to the integrity disruption of nanomedicine. Indicatively, some impacts of ultrasound-induced cavitation are high local forces that enhanced targeted delivery of drugs in nanodroplets 130. A specific method of delivery encapsulating the microRNA (antagomir-155) in LNPs was designed. The ultrasound was low-intensity and used to cause the gas vesicle (GV) cavitation which showed promising effectiveness in cardiac transplant rejection therapy 86. The collapse of ultrasound-induced GVs led to microflows and mechanical shear forces, which raise the vascular endothelial permeability to allow LNPs to extravasate into the myocardial tissue and extends cardiac allograft survival dramatically.

Nanomedicine based on magnetic response can be a major benefit in organ transplantation treatment of functional imaging, targeted drug delivery, and controlled and consistent heating. The nanomedicine can be directed to a transplantation site using magnetic guidance with improved and localized accumulation of immunosuppressive agents or imaging agents. The magnetic iron oxide nanoparticles have been used as an efficient MRI contrast agent and they provide noninvasive and real time division of the graft functionality and mapping of the nanomedicine so as to enhance the sensitivity and precision in postoperative assessment 131. Also, it is further proposed that, under alternating magnetic field (AMF), magnetic iron oxide nanoparticles produce localized heating and can be used to perform a focused thermal procedure without the dangers of employing the thermal temperature of an entire organ 132. Already, more sophisticated combined magnetic targeting, imaging, and nanowarming is being developed to provide complete transplant organ protection and monitoring. As an example, Robertson *et al.* used siRNA-conjugated magnetic nanoparticles to label the isle 90. Iron oxide nanoparticles (IONPs) can be used as effective nanowarming agents. When they are uniformly dispersed in organs via perfusion, IONPs generate heat at an equivalent level in the organ under an AMF, reducing ice crystal formation and thermal stress during rewarming. Gao *et al.* developed silica-coated IONPs (sIONPs) for homogeneous perfusion and effective clearance in rat kidneys 91. By coating commercial EMG308 IONPs with silica and modifying them with PEG and trimethoxysilane (TMS), the nanoparticles maintained a high heating efficiency under an AMF (360 kHz, 20 kA/m) while preserving colloidal stability in cryoprotectants. In rat kidney perfusion models, sIONPs were distributed evenly through glomerular capillaries without causing vascular obstruction, demonstrating sIONPs could be a promising low-damage nanowarming agent for transplant organs.

Although single-stimulus systems have been used to establish a basis of responsiveness, multi-stimuli-responsive protocols to improve targeting precision have been used with increasing frequency. Multi stimuli-responsive approaches have a valuable benefit in combining endogenous stimuli (pH, ROS and enzyme activity). The multi-stimulus systems will be in a position to deal with the overall heterogeneity of the environment following transplantation, thereby minimizing off-target release significantly. It is also possible to augment spatiotemporal accuracy of drug delivery through additional delivery of exogenous stimulus that may be magnetic fields, light or ultrasound and to reduce interindividual differences. Additionally, sequential response mechanisms which are reached through administration of several stimuli in a given sequence makes sure that there is site and stage specific activation of drugs at the end of a series of required biological responsive stages 137. Although that is early in the development of the mold of organ transplantation use, this strategy has a huge potential in precise therapy. To speed up the clinical translation of this system, the multi-stimuli system may be combined with particular targeting needs of transplant immunology to enhance it.

## 4. Advances in nanomedicine application for organ transplantation

In pretreatment, nanomedicines enhance graft quality through uniform nanoparticle-enabled rewarming, which reduces cryopreservation injury and IRI, as well as through integration with *ex vivo* perfusion systems. Perioperatively, nanocarriers deliver therapeutic agents to injury sites to clear reactive oxygen species and mitigate inflammation. In post-transplantation, nanomedicines enable targeted immunosuppression, improving treatment efficacy while minimizing off-target effects and supporting immune tolerance. Additionally, nanotechnology contributes to early diagnosis and theranostic applications, thereby facilitating continuous monitoring and adaptive treatment throughout the transplantation process (**Table 2**).

### 4.1 Nanomedicine for donor organ preconditioning

Donor organ pretreatment during the interval between procurement and transplantation is often conducted through mechanical perfusion, and nanomedicine can be incorporated into the perfusion process. The drug concentration within the target organ is elevated via the localized nanomedicine delivery approach and systemic circulation of the nanomedicine is circumvented, thereby reducing clearance and off-target effects 29. Since the donor organ remains isolated during perfusion, direct delivery of immunosuppressants in nanomedicine prevents systemic immune interference and contributes to a reduction in the reliance on postoperative high-dose immunosuppressive regimens 163.

The integration of immunosuppressant-loaded nanomedicine into NMP is currently actively explored. For instance, PEG-PLGA nanoparticles were employed to deliver mycophenolate mofetil (MMF) for donor heart pretreatment, successfully reducing posttransplant vascular lesions 133. MMF, an immunosuppressant, inhibits purine synthesis in lymphocytes via blockade of inosine monophosphate dehydrogenase (IMPDH), thereby suppressing T-cell proliferation and exerting an anti-endothelial cell activation effect. Direct musical in a model of murine heart transplantation saw the retention of the MMF-loaded nanomedicine in the donor heart, and delivered sustained release of the drug without immunological effect systemically in the spleen or lymph nodes. This gave major effects in attenuation of the early inflammation, vascular injury and fibrosis and better graft-long term survival. This *ex vivo* nanotechnology platform achieved localized immunosuppression to the graft site, thereby preventing chronic rejection and reducing systemic potential risks (e.g., infection and malignancy).

**Figure 5:**
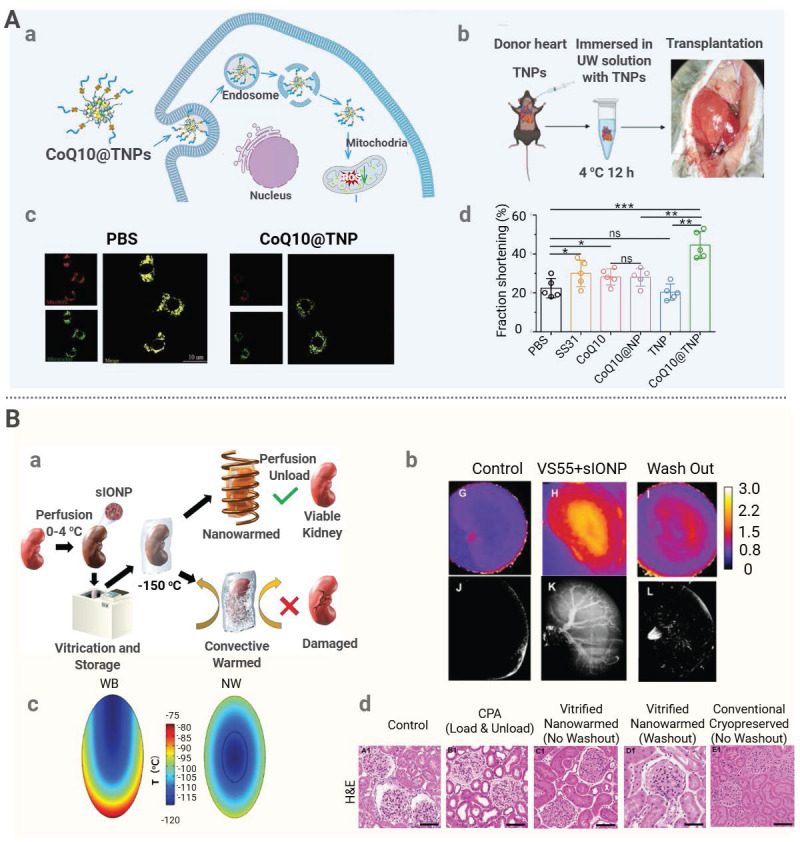
**Nanoparticle-based strategies for organ preconditioning.** (A) Graft pretreatment to attenuate IRI. (a) Scheme of CoQ10@TNPs. (b) Schematic of a donor heart (DH) perfused with CoQ10@TNPs. (c) The MtROS content in H9c2 cells measured by MitoSOX staining. (d) Ejection fraction and fractional shortening of the cardiac graft on day 1 post-transplantation. Adapted with permission from 135, copyright © 2025 Springer Nature. (B) (a) Schematic of the kidney nanowarming process. (b) MR and µCT images for the distribution of sIONPs, which was based on the water relaxation rate constant. (c) Temperature distribution within a kidney section. (e) H&E photomicrographs of kidneys. Adapted with permission from 139, Copyright © 1999-2025 John Wiley & Sons, Inc or related companies.

Yuan *et al.* developed a mitochondria-targeting nanomedicine system (CoQ10@TNPs) for donor heart pretreatment to mitigate IRI 134. A coenzyme, Q10 (CoQ10), was encapsulated in composite nanoparticles composed of calcium carbonate, calcium phosphate, and biotinylated carboxymethyl chitosan, and surface modification of the composite nanoparticles was performed by introducing a mitochondrial-targeting peptide, SS31, to achieve organelle-specific delivery. It was demonstrated that during *ex vivo* preservation, CoQ10@TNPs administered via aortic perfusion preferentially accumulated in the donor heart mitochondria, which led to a significant reduction in mitochondrial ROS production, a decrease in oxidative damage, and suppression of apoptosis and inflammatory responses, ultimately improving the post-transplant cardiac function (**Figure 5A**). Another novel strategy was applied during donor organ preservation to avoid systemic immunosuppression by engineering the vascular endothelium of the donor organ with a glycopolymer, LPG-Q-Sia3Lac, during cold preservation. Through tissue transglutaminase-mediated conjugation, the polymer formed a stable barrier on the endothelial surface. The sialic acid residues on this barrier bound to Siglec receptors on immune cells, thereby suppressing the activation of NK and CD8^+^ T cells. This treatment significantly reduced early inflammation as well as acute and chronic rejection in aortic transplant models, while in kidney transplantation models, it markedly alleviated IRI and improved the renal function 164.

Nanomedicine can be employed for donor organ preconditioning during the rewarming phase after vitrification 165. Magnetic nanoparticles, such as SPIONs or sIONPs, can be preinfused into organ vasculature prior to cryopreservation. Under an AMF, these nanoparticles generate internal heat for rapid and uniform rewarming, preventing ice crystal damage 166. This approach has been validated across multiple biological models. For instance, a magnetic cryoprotectant (mCPA) composed of PEG-coated SPIONs and VS55 was developed 135. This mCPA formulation achieved a heating rate of 321 °C per minute, far exceeding 50 °C per minute required for vitrification. Rat hearts cryopreserved for one week were able to fully recover structurally and functionally after mCPA-assisted rewarming. In another study, pancreatic islets were rewarmed with SPIONs at 5 mg/mL under an AMF and a warming rate of 72 °C per minute was achieved 136. After transplantation into diabetic mice, these islets restored normoglycemia, whereas those thawed by water bath failed. These findings suggest nanowarming could enhance the tissue quality and improve the transplantation of complex tissues.

Additionally, a silica shell of sIONPs mitigates particle aggregation and sIONPs have low immunogenicity. Han *et al.* successfully conducted kidney transplantation in a rat model after the application of PEG-modified sIONPs in conjunction with a low-toxicity cryoprotectant, VMP 137. It was shown that after sIONPs were uniformly distribution in the kidney, uniform and rapid internal rewarming of the organ was achieved under an AMF. The kidney cryopreserved up to 100 days regained its normal function after nanowarming. The key renal function indicators remained stable over a 30-day post-transplantation period, confirming the graft viability comparable to freshly transplanted kidneys. Similar results were obtained by Sharma *et al.* when they administered sIONPs in conjunction with a cryoprotectant into the kidney via vascular perfusion. Microcomputed tomography (μCT) and MRI confirmed uniform distribution of nanoparticles within the renal vasculature and both techniques were also used for real-time monitoring of organ vitrification 138. Histological analysis revealed that the kidney cells in the nanoparticle-rewarmed group exhibited a high viability and preserved the endothelial structure, comparable to those in kidneys perfused with cryoprotectants alone without cryopreservation or rewarming (**Figure 5B**). The research team also investigated the efficacy of sIONPs in liver rewarming after cryopreservation. Under an AMF, sIONPs were stimulated to generate rapid (61 °C/min) and uniform heating. The rewarmed liver showed an intact tissue structure, preserved vascular endothelium, and substantially recovered the hepatocyte function 139. Despite mild impairment for bile excretion and a slight increase in the alanine aminotransferase (ALT) level, the overall damage to the treated organ was minimal.

These findings confirm that nanomedicine-based interventions during the critical pretransplant period can enhance the graft quality through targeted drug delivery or uniform and rapid rewarming and enable localized immunomodulation. This innovative nanomedicine-based platform could remarkably improve transplantation outcomes. However, it should be noted that current proof-of-studies are predominantly confined to *ex vivo* rodent models, and it is essential to verify these findings from rodent models in larger animal models and eventually on human donor organs to establish clinical relevance and readiness for translation.

### 4.2 Nanomedicine for ischemia-reperfusion injury intervention

IRI is characterized by hypoxic damage to organ during ischemia and exacerbated by the initiation of oxidative stress, inflammatory responses, and apoptosis during reperfusion. IRI can adversely impair the graft functionality. Owing to its precision delivery through active targeting, nanomedicine can load and transport antioxidant enzymes to specific target sites, thereby directly neutralizing substantial ROS generated during reperfusion and effectively alleviating oxidative damage 167. For example, Yan *et al.* developed a nanozyme drug, n(SOD-CAT), composed of superoxide dismutase (SOD) and catalase (CAT), to mitigate IRI during liver transplantation 140. Negatively charged SOD and CAT were electrostatically self-assembled with a cationic polymer, PEG-g-BPAH, to form an initial complex, P(SOD-CAT). Subsequent surface-initiated atom transfer radical polymerization of the complex yielded crosslinked shells, leading to the formation of n(SOD-CAT). Under a physiological condition, the nanomedicine maintained a positive surface charge that promoted hepatocyte uptake. In a murine model of hepatic IRI, treatment with n(SOD-CAT) significantly reduced the serum levels of AST, ALT, and TNF-α compared to free enzymes, and attenuated histopathological damage and apoptosis. This nanoplatform allows efficient enzyme co-delivery and targeted therapy, and it could be used as a novel approach to managing IRI during transplantation.

**Figure 6:**
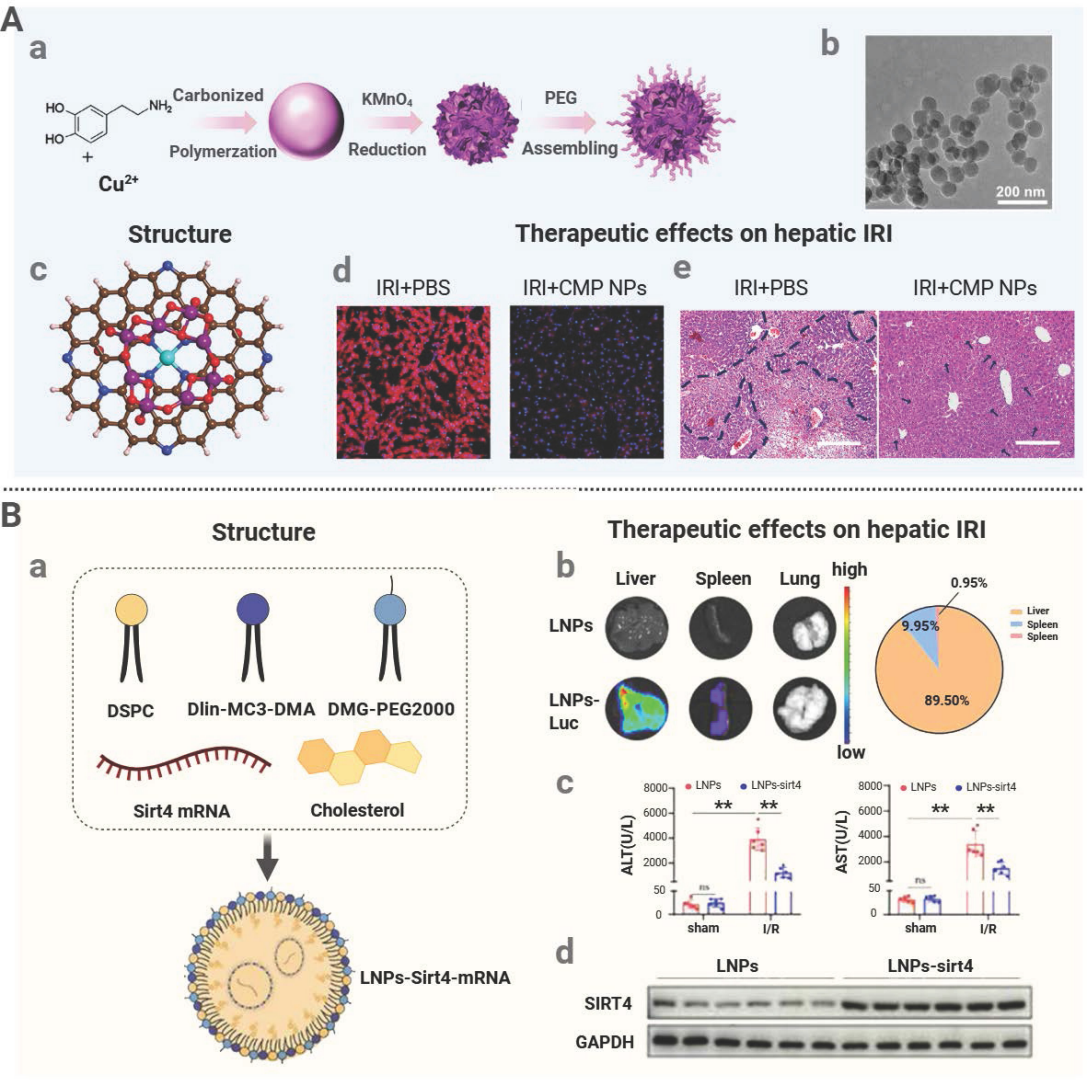
**Nanomedicines against IRI.** (A) An antioxidant nanoenzyme against IRI. (a) Synthesis route of CMP NPs. (b) TEM characterization images of Cu_us_-pC. (c) The optimized structure of Cu_us_-pC@MnO_2_. The color for each element: C: brown; N: blue; Mn: purple; O: red; H: pink; Cu: cyan. (d) DHE staining of liver tissues. (e) H&E staining of liver tissues from each group. Adapted with permission from 142, Copyright © 1999-2025 John Wiley & Sons, Inc or related companies. (B) (a) Scheme of LNPs-Sirt4. (b) The expression of LNPs-Sirt4 in the liver, spleen, and lung. (c) Serum ALT and AST levels in different groups of mice. (d) Western blot analysis of SIRT4 protein expression in the livers. Adapted with permission from 63, Copyright ©2025 Ivyspring International Publisher.

In addition to delivering natural enzymes, nanozymes have gained significant attention for IRI treatment. These nanomaterials exhibit enzyme-like catalytic properties. By emulating the catalytic function of natural enzymes, such as SOD and CAT, nanozymes can effectively scavenge free radicals and mitigate oxidative stress 168. For instance, Lin *et al.* developed an ultrasmall copper-based nanozyme (Cu_us_·pC@MnO₂@PEG) for mitigating mouse liver IRI (**Figure 6A**). The nanozyme was synthesized through carbonization and subsequent redox reaction to form an optimized structure. The Cu-N₄ sites exhibited the SOD-like activity to convert superoxide into H₂O₂, and the MnO₂ outer layer displayed the CAT-like activity to decompose H₂O₂ into oxygen and water. In a murine IRI model, this nanozyme significantly lowered serum AST and ALT levels, restored the SOD activity and increased the ATP content in the liver tissue, and reduced ROS accumulation and diminished hepatocyte necrosis 141.

Nanomedicine has been shown to significantly enhance therapeutic efficacy against IRI by enhancing targetability, bioavailability, and biocompatibility of anti-inflammatory and antioxidant agents. For instance, a synthetic bilirubin-PEG conjugate (BX-001 N) self-assembled into uniform-sized nanoparticles. The ROS-scavenging capacity of bilirubin 3α was preserved in the nanoparticles, while its inherent limitations, such as oxidative instability and poor solubility, were overcome. When applied in a model of renal IRI, BX-001 N was more effective and less toxic compared to free bilirubin 142. In the second study of mitigating hepatic IRI, neutrophil membrane-coated taurine-loaded mesoporous silica nanoparticles (nanotaurine) were deposited in the damaged tissue through companion with ICAM-1. This preparation had a far-reaching effect on decreasing the concentration of liver injury markers and inflammatory edibles and increasing the antioxidant action in the mice 143. The current progress in metal-organic framework (MOF) hydrogels has enabled the emergence of a novel approach to the prevention and treatment of IRI following organ transplantation. An example of this kind of MOF hydrogel is the one generated by Zhou *et al.* as a MOF hydrogel (MOFSP) that had Rhodiola glycosides 144. A PEG-modified UiO-66-NH_2_ structure was entraped into a 5-hydrogel of 5 g-polyglutamic acid/oxidized sodium alginate (5 -PGA/OSA) in this design. This composite attached well to the heart, where it got prolonged release of the drug and better distribution of drug in the heart organ. The nanoplatform obtained by MOF may be potential in management of myocardial IRI.

Nanomedicine has the potential to alleviate IRI by regulating the metabolic activities of neutrophils selectively to avoid imprudent activation. Neutrophils mediate IRI pathogenesis, releasing proinflammatory mediators and creating neutrophil extracellular traps (neutrophil extracellular traps (NETs) which is mainly regulated by mitochondrial ROS 169. To exploit this process, Lu *et al.* harvested exosomes generated by mesenchymal stem cells of the human umbilical cord (hUC-MSC-EVs) to transfer functional mitochondria to hepatic neutrophils and thereby benefited the reinstatement of the mitochondrial integrity and inhibition of the NET formation 145. The number of these exosomes containing intact mitochondria was approximated to be 40 percent, and these mitochondria were vesicular and were transfer of the mitochondria occurred through membrane fusion with neutrophils hence stabilizing the mitochondrial membrane potential. The treatment of neutrophils in a mouse model of liver IRI with hUC-MSC-EVs stimulated a glycolysis-oxidative phosphorylation transition, demonstrated by a reduced lactate, which hence restored the NAD^+^/NADH ratio. This reprogramming of metabolism blocked net release and production of the proinflammatory cytokines, which eventually lessened tissue injury in the liver. In a comparable method of modulating metabolism, liposomal nanoparticle (NP-Ly6G(2-DG)) was temporarily functionalized with an anti-Ly6G antibody in the delivery of 2-deoxy-D-glucose specifically to neutrophils 146. Competitive inhibition of hexokinase and phosphoglucose isomerase by the nanoparticle neutralized the glycolytic activity and caused severe energy decrease, which subsequently inhibited the NET development and enhanced neutrophil apoptosis. This metabolic intervention, which is targeted, reduced pulmonary damages and enhanced oxygenation in a lung IRI model.

Gene therapy is a type of therapy that regulates or manipulates the expression of the genes via injection of the nucleic acid-based pharmaceuticals to affect the target cells and has demonstrated significant potential in IRI treatment 170. The most important part of this modality is the LNPs which are effective in capturing various types of nucleic acids, i.e. mRNA, siRNA, and plasmid DNA. This encapsulation process significantly limits the danger of nucleic acid breakdown due to enzymatic activities of nucleases, which maintain the therapeutic intact nature of nucleic acids-based medications. In the given example, Yu *et al.* developed a liver-specific LNP formulation and encapsulated the SIRT4 mRNA to create LNP-sirt4 mRNA to counteract hepatic IRI 63. The transfected SIRT4 mRNA was then translated into SIRT4 protein, which is a mitochondrial deacetylase and interacts with PRDX3 to facilitate deacetylation of PRDX3. This therapy prevented ferroptosis, hepatocyte-apoptosis, and serum- ALT and AST concentrations all significantly, therefore showing a clear therapeutic impact on hepatic IRI (**Figure 6B**).

Overall, nanomedicine has the potential of decreasing oxidative stress and mitigating cell death, by delivering antioxidants, enzyme mimic, anti-inflammatory agents, and modulators of metabolic processes into the body to eliminate ROS and destabilize inflammatory and metabolic processes. Moreover, the mechanism of regulation of genes can be reached with the help of targeted delivery of nucleic acid with the use of nanomedicine to reduce IRI during organ transplantation. The IRI pathophysiology is yet to be revealed and improved nanoplatforms may be recognized on the basis of the revealed IRI pathophysiology to cooperatively affect the target pathways of injury. However, it is noteworthy that the vast majority of current nanomedicine studies exploit one single individual fundamental mechanism of IRI, while validation and adaptation within complex transplantation models remains to be addressed. More translational studies should be conducted to bridge mechanistic findings with transplant-specific applications.

### 4.3 Nanomedicine for anti-rejection therapy

Nanomedicine can enhance immunosuppressive efficacy during organ transplantation through targeted delivery of immunosuppressants to critical immune cells and modulating essential pathways, realizing rapid and efficient immune suppression. Moreover, strategies of lymphoid organ targeting and localized immunomodulation can be employed to promote immune tolerance of implants and support long-term graft survival.

Nanocarriers can be used for targeted delivery of immunosuppressants, such as tacrolimus and rapamycin, to immune organs or cells, to reduce their systemic exposure and mitigate immunosuppressant-related adverse effects. For instance, the mPEG-PLGA-PLL (PEAL) backbone on an engineered nanoparticle (aNP) was conjugated with an anti-IL-21R antibody for precise tacrolimus delivery 147. Since IL-21R is predominantly expressed on T follicular helper (Tfh) and B cells in lymphoid organs, aNPs abundantly accumulated in the spleen and lymph nodes to increase the local tacrolimus concentration. Meanwhile, the antibody blocked IL-21/IL-21R signaling, thus inhibiting CD4^+^ T cell differentiation into Tfh cells. In a murine skin graft model, aNP treatment suppressed T- and B-cell responses, preserved the graft tissue integrity, reduced lymphocyte infiltration, prevented acute rejection, and lowered tacrolimus-induced nephrotoxicity. In another study, a pH-responsive nanomedicine containing tacrolimus (Tac-NP-CD4Ab) was developed to target CD4^+^ T cells via an anti-CD4 antibody and enhance its accumulation in the spleen via a spermine-modified acetylated dextran coating on the mesoporous silica core of the nanomedicine 148. Upon lysosomal acidification, released tacrolimus suppressed T follicular helper cell differentiation, thereby inhibiting downstream plasma cell formation and donor-specific antibody production. This targeted delivery mechanism not only improved renal histology and function after transplantation but also markedly reduced nephrotoxicity, metabolic disorders, and neurotoxicity which are often seen during conventional systemic administration of tacrolimus. In addition to the use of nanocarriers, an alternative strategy is to directly apply poorly soluble nano-sized drugs, and injectability and stability of these nanodrugs in aqueous solutions can avoid systemic toxicity associated with high-dose oral administration. Recently, self-assembly of RAPA into nanoparticles was realized under an ultrasonic condition (120 W) without any exogenous carriers or stabilizers 172. The resulting nanoparticles exhibited favorable stability, biocompatibility, and immunosuppressive effects, effectively addressing poor water solubility and low bioavailability of RAPA.

Nanotherapeutics offer a promising approach to modulating inflammatory responses by accomplishing targeted delivery of immunosuppressive agents and selective elimination of specific immune populations. A representative example is the Ce6-NP-MCP-1 system developed by Li *et al.* This nanosystem integrated CCR2-directed targeting with photodynamic therapy to mitigate acute rejection in heart transplantation 149. To construct this nanosystem, a PEG-PLGA carrier was used to encapsulate a photosensitizer, chlorin e6 (Ce6), and its surface was functionalized with MCP-1 peptide to promote selective uptake by CCR2-positive macrophages in the graft. Upon internalization and subsequent exposure to low-intensity ultrasound, Ce6 was stimulated to generate ROS and induce apoptosis in the host macrophages. The targeted intervention reduced inflammatory infiltration in cardiac allografts and prolonged graft survival, suggesting the localized delivery strategy could be promising for immunomodulation during transplantation.

**Figure 7:**
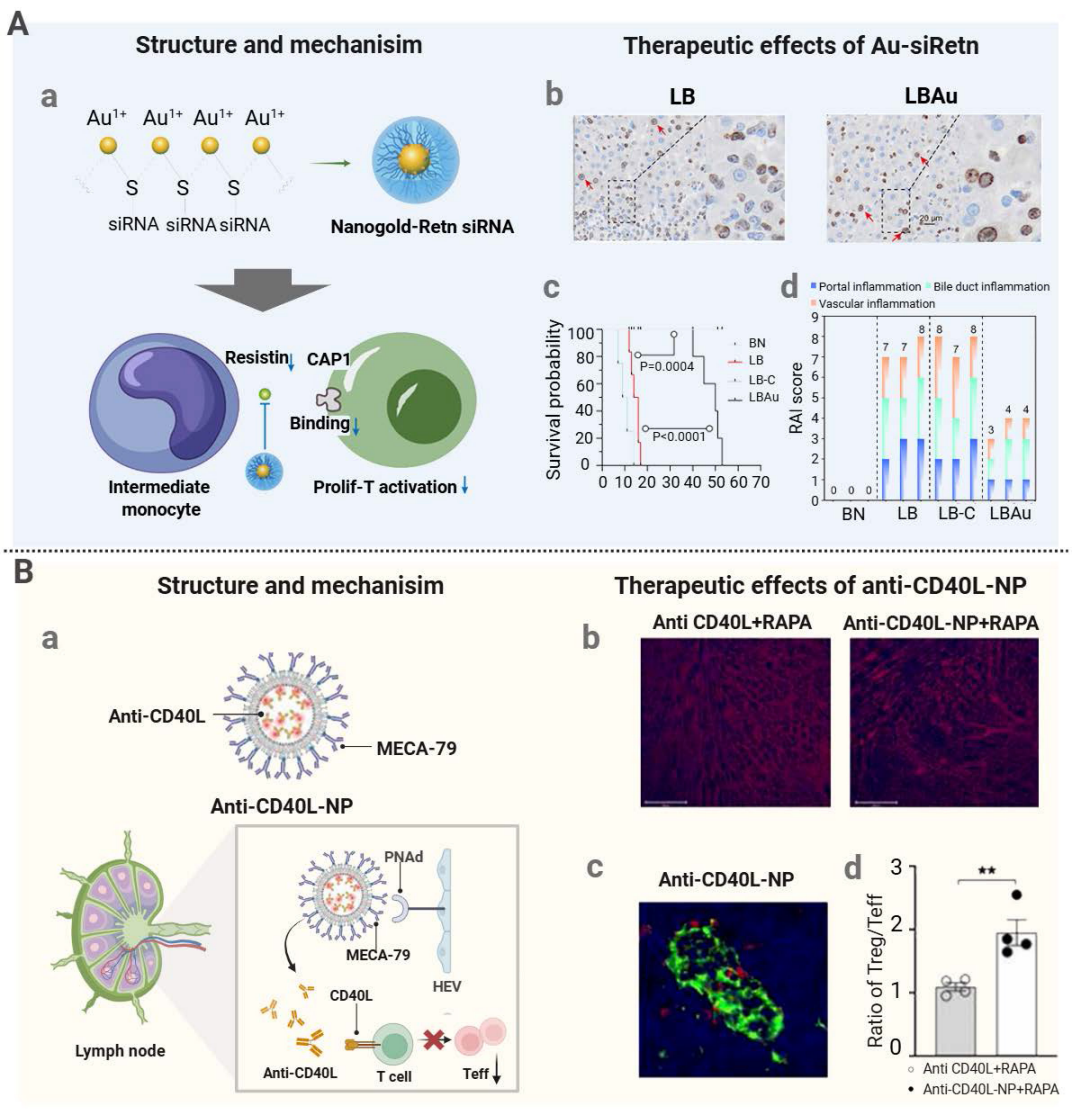
**Nanotherapies for post-transplant rejection.** (A) (a) Scheme of Au-siRetn. (b) Hepatocyte proliferation detected by Ki67 staining. (c) Survival curve analysis of recipients. (d) Statistical analysis of the RAI score of recipients. Adapted with permission from 151, Copyright © 2025 The Authors. (B) (a) Scheme of anti-CD40L-NPs. (b) Representative fluorescence micrographs of fibronectin staining in heart allograft sections of WT recipients. (c) Immuno fluorescent staining of HEVs of DLNs from mice treated with anti-CD40L. (d) Comparison of the Treg/Teff ratio. Adapted with permission from 157, Copyright © 2025 American Society for Clinical Investigation.

Besides, nanomedicine will be able to find particular genetic pathways to fight organ transplant rejection. Yang *et al.* found that intermediate monocytes secreted to stimulate T-cell proliferation and activation worsened the cases of rejection in liver transplant recipients (**Figure 7A**). Resistin interacts with proliferative T cells, especially overexpressing CAP1 gene which worsens rejection. To reduce resistin secretion, a gold nanoparticle-based siRNA delivery system (AU-siRetn) was developed to achieve targeted knockdown of the Retn gene in a rat model for allogeneic liver transplantation 150. The results revealed that AU-siRetn significantly decreased the resistin expression level, reduced T-cell proliferation in the liver, alleviated portal vein inflammation, bile duct inflammation, and vascular endothelial damage, and notably prolonged liver graft survival. In addition to the suppression of the TMBR, nanotechnology-based gene therapeutic strategies have been explored for the mitigation of the AMBR. For instance, C5 siRNA-loaded LNPs were developed to suppress complement activation during renal transplantation 151. The LNPs were synthesized from ionizable lipids, DSPC, cholesterol, and PEG-lipids via microfluidics, and they were employed to deliver siRNA targeting complement component C5. After PEG detachment upon arrival at the graft site, apolipoprotein E adsorption mediated hepatocyte-specific uptake through LDL receptors. In a sensitized rat kidney transplant model, the combination of C5 siRNA-LNPs and RAPA significantly prolonged graft survival and improved the renal function. This is the first application of RNAi for complement inhibition in antibody-mediated rejection. Although the treatment with C5 siRNA-LNPs enhanced graft survival, more durable silencing strategies should be developed for persistent chronic rejection histopathology. This study underscores broad challenges of controlling chronic graft deterioration despite effective management of acute rejection.

Immune tolerance is a condition of antigen-specific immune unresponsiveness. In the field of organ transplantation, the induction of immune tolerance is a highly specific and long-lasting novel therapy to eliminate patients from lifelong consumption of perpetual immunosuppressive drugs 171. Regulatory T cells (Tregs) induce immune tolerance by exerting active suppression of effector T-cell immune response. Therefore, enhancing the in vivo quantity or function of Tregs has become a common strategy to improve immune tolerance 172. RAPA can inhibit T-cell activation and proliferation through blockade of the transition from the G1 to S phase via inhibition of mTOR pathway, while low-dose RAPA promotes Treg expansion via partial mTOR suppression. This redirects native T-cell metabolism toward oxidative phosphorylation, fostering Treg survival, which helps maintain an immature, tolerogenic state of T cells when they are in contact with APCs. Expression of costimulatory molecules is suppressed to induce T-cell dysfunction or block their differentiation into effector T cells 173. Based on the above research findings, a rapamycin-loaded PEG-PPS nanopolymer (rPS) was prepared for subcutaneous injection. The drug was absorbed through the lymphatic vessels and accumulated in the lymph nodes rich in APCs 152. In a murine islet transplantation model, rPS inhibited T-cell proliferation and normoglycemia was sustained for over 100 days, significantly prolonging graft survival. In another study, Fas ligand (FasL)-presenting PLGA nanoparticles loaded with rapamycin (FasL@Rapa NPs) were engineered 153. These nanoparticles induced T-cell apoptosis through FasL-Fas interaction and expanded Treg through the sustained release of RAPA. Upon co-transplantation with islets, they suppressed CD8⁺ T cells and enhanced the Treg activity, significantly extending allograft survival. In contrast to RAPA, BEZ235, a second-generation mTOR inhibitor, can completely block the phosphatidylinositol 3-kinase (PI3K)-AKT-mTOR signaling pathway, resulting in more robust inhibitory effects on effector T-cell activation and proliferation and greater stimulatory effects on Treg expansion 174. Xing *et al.* developed chitosan-based nanoparticles (BEZ235@NPs) to encapsulate hydrophobic BEZ235. Its permeation was enhanced through phospholipid bilayers and its intracellular drug concentration was increased within T cells 154. The experimental data supported that BEZ235@NP significantly increased the survival time of mouse cardiac grafts. It weakened CD4⁺ and CD8⁺ T-cell activation and infiltration, while it markedly increased the Treg proportion, thus confirming BEZ235 could be applied to induce posttransplant immune tolerance. Che *et al.* developed hybrid calcium carbonate/calcium phosphate/heparin nanoparticles loaded with fingolimod (FTY720) and surface-modified the nanoparticles with a CCL21 ligand (CCL21#6.10R) for lymph node-targeting delivery 155. In a cardiac transplant model, these nanoparticles selectively released FTY720 in lymph nodes, and FTY720 bound to sphingosine-1-phosphate receptor 1 (S1P1R) on lymphocytes to inhibit T-cell activation and migration. Simultaneously, CCL21#6.10R engaged CCL21 on CCR7-positive cells to attenuate effector T-cell responses and intensify immune tolerance. In the treated mice, peripheral T-cell counts decreased significantly, while the regulatory T-cell to effector T-cell ratio in lymph nodes remarkably increased, ultimately leading to long-term graft survival.

In the context of immunomodulatory strategies, nanomedicine can be designed to target and inhibit CD40 on the surface of T lymphocytes, thus obstructing costimulatory pathways critical for T-cell activation. This method does not only subdue early activation of T-cells, but it also aids the variousiation of T-cells into Tregs. Zhao and others pioneer the designing of a new type of nanoparticles which is made of PLGA in order to carry in and release anti-CD40L antibodies. The MECA-79 monoclonal antibodies on the nanoparticle surface were conjugated with covalent DCS to increase immunological tolerance in a cardiac transplantation model in a mouse 156. MECA-79 permits peripheral lymph node addressins (PNAd), to be targeted to the lymphatic system because of its specificity to the density lipoprotein receptor. Upon release, the anti-CD40L antibodies bound to CD40 on DCs to disrupt its interaction with CD40L on T cells and impede the acquisition of effective costimulatory signals by T cells. When these nanoparticles were combined with RAPA, a robust synergistic effect was observed, and the survival of the heart grafts was extended to 80 days (**Figure 7B**).

In addition to inducing T-cell differentiation toward Tregs by blocking initial costimulatory pathways, nanomedicine can be employed to foster an immunosuppressive microenvironment conducive to long-term graft survival without the need for continuous potent interventions after implantation. Liu *et al.* have demonstrated that konjac glucomannan-modified silica nanoparticles (KSINPs) remodeled the splenic architecture in both mice and nonhuman primates, and transformed the remodeled spleen into an optimal site for transplantation that supported prolonged survival of allogeneic or xenogeneic islets 157. KSINPs effectively targeted splenic macrophages via the affinity of konjac glucomannan to mannose receptors, inducing receptor clustering and promoting M2 polarization. Simultaneously, silica-mediated activation of TGF-β signaling stimulated fibroblast proliferation and extracellular matrix (ECM) production while suppressing immune cell infiltration. In murine and nonhuman primate models of islet transplantation into KSINP-remodeled spleens, grafts showed prolonged survival and sustained insulin secretion. In another similar study, a co-transplantation system was developed for type 1 diabetes 177. The system sustained the islet function within a supportive artificial microenvironment. RAPA-loaded β-cyclodextrin nanoparticles were covalently anchored onto islet-laden GelMA microgels, and sustained drug release for over 30 days was achieved. The microgel structure provided physical isolation of islets, effectively shielding them from immune effector cells. Furthermore, co-transplanted oxygen-generating microspheres locally alleviated hypoxia prior to revascularization. In diabetic mice, this strategy maintained normoglycemia for 90 days, demonstrating promising therapeutic potential.

Distinctive advantages of nanomedicine have been demonstrated in the domain of transplant rejection management. Nanomedicine can enhance therapeutic efficacy while minimizing systemic toxicity by precisely delivering immunosuppressants to lymphoid organs or immune cells. Furthermore, targeted gene regulation, Treg expansion induction, and the establishment of an immunosuppressive microenvironment via multifunctional nanomedicine facilitate effective and sustained modulation of immune responses. Although the expansion of Tregs represents one of the most critical pathways to establish immune tolerance, the definitive evidence of immune tolerance in experimental models should be consolidated by long-term graft survival after complete withdrawal of immunosuppressive agents. Clinical translation of nanomedicine for transplant rejection management should be accelerated and innovative nanomedicine approaches could be explored for the management of chronic rejection and precise subcellular delivery.

### 4.4 Nanomedicine for postoperative monitoring and treatment evaluation

**Figure 8:**
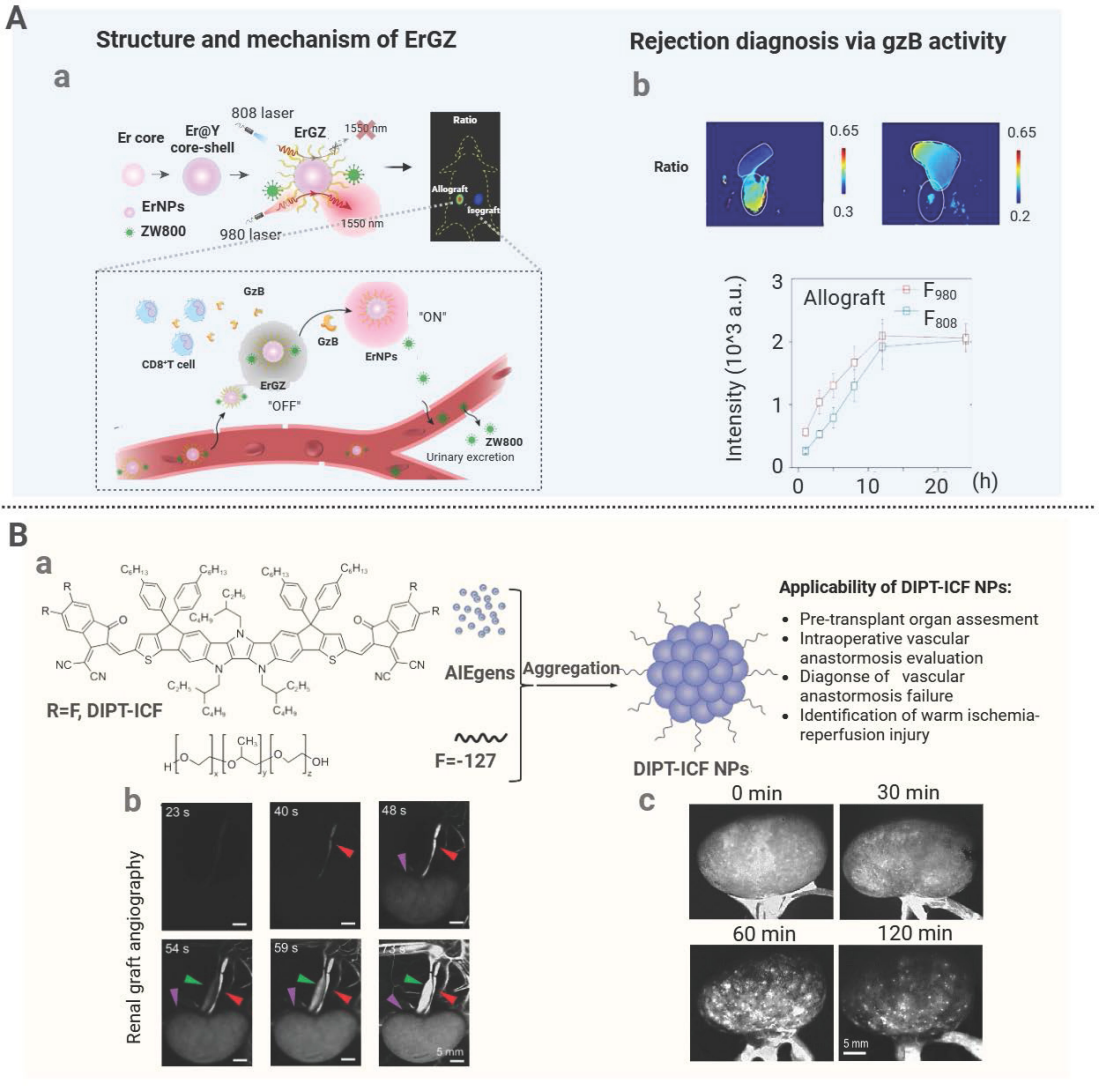
**Nanoprobes for noninvasive diagnosis of transplantation.** (A) (a) Scheme of a GzB-responsive nanosensor. (b) *In vivo* lateral view of NIR-II fluorescence ratio images and intensity on mice received islets transplantation. Adapted with permission from 160, Copyright © 1999-2025 John Wiley & Sons, Inc or related companies. (B) Dynamic monitoring of kidney transplantation. (a) Chemical structures of DIPT-IC, DIPT-ICF and amphiphilic polymer F127. (b) NIR-II images of graft arteries, veins and graft reperfusion. (c) NIR-II images of kidneys under different warm ischemia durations. Adapted with permission from 162, Copyright © 2025 Oxford University Press.

Nanomedicine has been explored for diagnosing posttransplant rejection. A recently developed granzyme B (GzmB)-responsive nanosensor (GBRN) was used for noninvasive rejection monitoring in a murine heart transplant model 158. GBRN was prepared from gold nanoclusters and surface-functionalized with GzmB-cleavable peptides. During rejection, GzmB at an elevated level in the graft cleaved these peptides on the surface of GBRN to release small gold nanoclusters. These filtered nanoclusters accumulated in urine and exhibited the peroxidase-like activity to produce a colorimetric signal upon addition of H₂O₂ and tetramethylbenzidine (TMB) for visual readout. *In vivo* studies confirmed that the urinary signal intensity was well correlated with cardiac dysfunction during rejection. The signal intensity was weakened after FK506 treatment but strengthened after its withdrawal. Notably, GBRN could detect the elevated urinary catalytic activity even at an early rejection stage when the graft contractility remained normal, supporting its sensitivity for diagnosing rejection prior to functional impairment. Another near-infrared-II (NIR-II) fluorescent nanosensor (ErGZ) was developed for early rejection diagnosis in skin and islet transplantation models 159. The sensor consisted of erbium-doped sodium fluoride nanoparticles (ErNPs) emitting at 1550 nm under dual-wavelength excitation. Its surface was conjugated with a GzmB-cleavable peptide linked to a quencher, ZW800. In the absence of GzmB, the fluorescence signal remained quenched. During the rejection process, GzmB was gradually elevated to a level sufficient for cleaving the peptide on ErNPs to release ZW800 and restore the ErNP fluorescence signal. The fluorescence intensity ratio under 808 nm and 980 nm excitation (F₈₀₈/F₉₈₀) could be used for real-time monitoring of variations in the GzmB activity in grafts, and released ZW800 could be detected in the urine. The ErGZ biosensor detected rejection 2 and 5 days earlier than histology in skin and islet transplant models, respectively, demonstrating its high sensitivity and specificity for early noninvasive diagnosis (**Figure 8A**).

During graft rejection, infiltrating macrophages display a proinflammatory M1 phenotype and release inflammatory cytokines to elevate the ROS level including H₂O₂, thereby amplifying inflammatory responses and inducing tissue damage. To exploit this mechanism, a macrophage-targeting H₂O₂-activated aggregation-induced emission (AIE) nanoprobe (MTBPB GPs) was developed for detection of early acute rejection by responding to H₂O₂ at an elevated level in M1 macrophages. The fluorescence signal was intensified from day 3 post-skin transplantation and the changes in the fluorescence signal were in line with the immunosuppressive efficacy 160. The AIE property helped enhance the fluorescence signal intensity during accumulation of the nanoprobe in macrophages, improving the detection sensitivity 175. Another NIR-II nanoprobe with the AIE property was developed for real-time fluorescence monitoring during kidney transplantation 161. An AIE molecule, DIPT-ICF, with a fused-ring electron acceptor (FREA) structure, exhibited intense and photostable NIR-II fluorescence signals in its aggregated state. The molecule was formulated with the F-127 surfactant to produce water-soluble nanoparticles, and these nanoparticles displayed prolonged circulation following intravenous administration. The nanoprobe derived from the nanoparticles enabled real-time visualization of renal arteries, veins, and their branching vasculature. In a rabbit transplantation model, the probe distinguishably delineated graft vascular structures, facilitated evaluation of urinary anastomosis patency, as well as detected surgical complications including vascular stenosis and embolism. Analysis of the fluorescence distribution pattern allowed assessment of the glomerular filtration barrier integrity and the IRI injury severity (**Figure 8B**). Additionally, a ROS-responsive polymeric probe (APNSO₂) was developed for early diagnosis of hepatic IRI 162. Upon cleavage by superoxide anion, the probe released fluorescent fragments detectable *in vivo* and in the urine within 1 h post-reperfusion, which was much earlier than the detectable time for conventional serum or histologic markers. Collectively, these nanoscale systems have been demonstrated to achieve noninvasive, sensitive monitoring of transplant-associated injuries.

In recent years, nanoplatforms by integrating diagnosis and therapy have been applied to the domain of organ transplantation. Robertson *et al.* engineered iron oxide nanoparticles by covalently functionalizing these nanoparticles with siRNA and encapsulating them with dextran to produce the MN-siRNAs nanomedicine. MN-siRNAs conferred protection to transplanted islets against apoptosis via caspase-3 gene silencing. Meanwhile, owing to the superparamagnetic property of iron oxide nanoparticles, MN-siRNAs could be used as an MRI nanoprobe to noninvasively monitor the graft post-transplantation 90. The islets were co-incubated with MN-siRNAs prior to transplantation. Upon transplantation of the labeled islets into a baboon model, MRI allowed dynamic and longitudinal tracking of the number of transplanted islets, thus performing noninvasive *in vivo* evaluation of siRNA-mediated antiapoptotic effects. This integrated diagnostic and therapeutic approach could be clinically translatable since it can be tailored for personalized treatment and long-term surveillance in diabetic islet transplantation.

Microenvironment-responsive nanoprobes have been demonstrated to achieve detection of the immune cell activity and the level of oxidative stress at the molecular level at the early phase of post transplantation, and they are superior over traditional detection methods in terms of diagnostic sensitivity and timeliness. Meanwhile, the integrated diagnostic and therapeutic nanoplatform combines interventional therapy with noninvasive monitoring, and it can deliver dynamic and timely feedback for postoperative management. However, currently developed diagnostic tools are predominantly used for monitoring the post-transplant organ status, and there are very few nanoprobes specifically designed for pre-transplant donor quality assessment. Sensitive, accurate nanoprobes should be developed for detecting early warning signals of chronic rejection, meanwhile, multimodal monitoring nanosystems could be explored for real-time monitoring of the transplantation process via detecting different biomarkers or metabolites to prolong graft survival.

## 5. Clinical translation challenges and future prospectives

**Figure 9:**
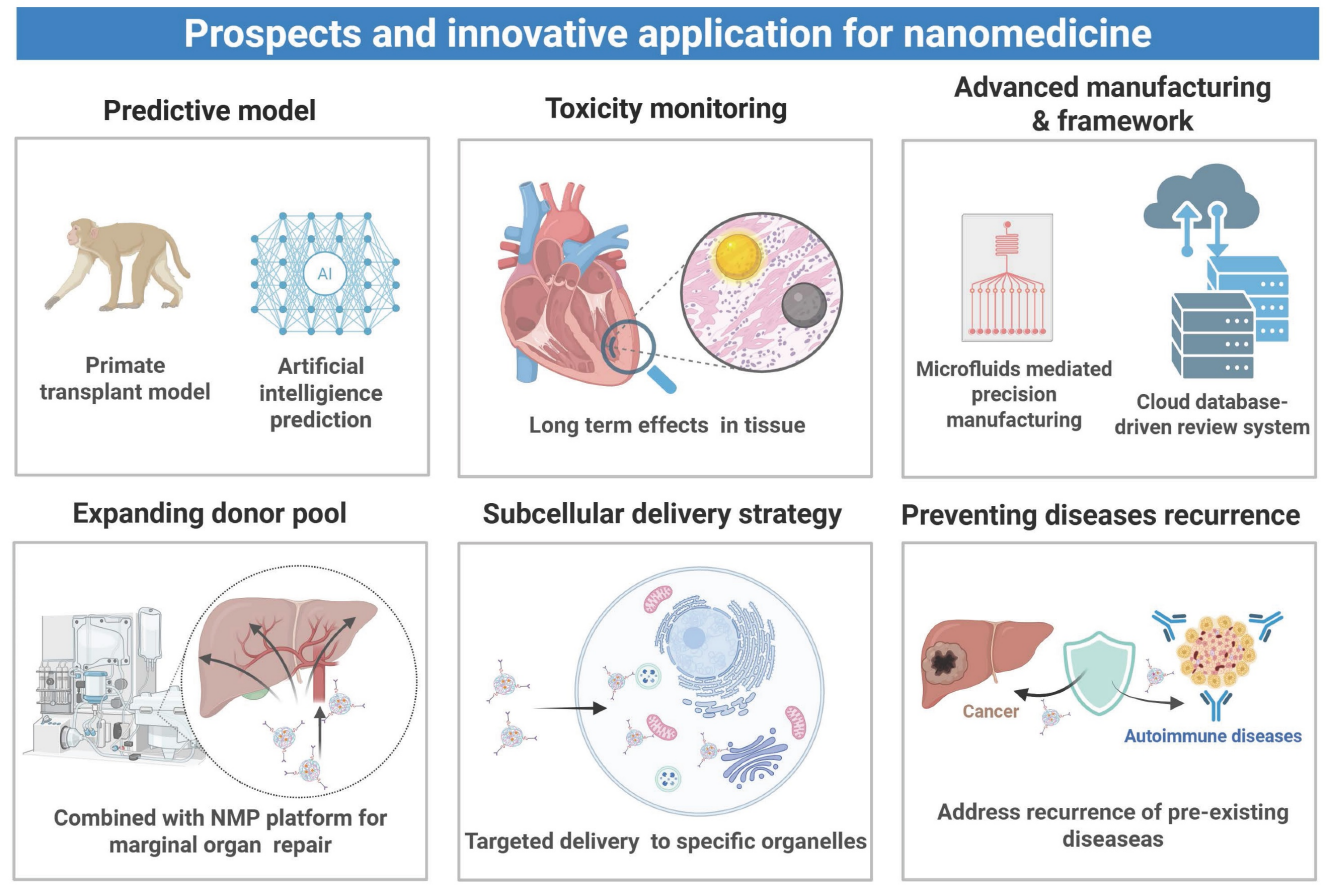
**Future directions in transplant nanomedicine.** Progress in accurate predictive models, real-time safety monitoring, and advanced manufacturing technologies in couple with coordinated review systems could accelerate clinical translation of nanomedicine for organ transplantation. Meanwhile, discoveries and innovations will expand the donor availability, achieve subcellular-level delivery, and prevent post-transplant disease recurrence in post-transplant recipients. Created with BioRender.com

Nanomedicine has been extensively explored in the field of organ transplantation at various stages, including organ protection, IRI mitigation, suppression of immune rejection, and posttransplant monitoring. However, the application of nanotechnology in transplantation faces notable challenges, particularly in the development of diagnostic tools. Currently, there are no effective non-invasive methods for evaluating donor organ quality. Although nanoprobes show potential for detecting organ dysfunction and may be useful for assessing donor quality, their safety and feasibility should be comprehensively evaluated before their clinical translation. During the formulation of nanomedicine strategies, most studies explore the responses to one single stimulus without considering integrated multi-stimuli systems, while stimuli-responsive approaches have been demonstrated for intelligent controlled drug release. The strategies of harnessing single stimulus lack operational flexibility and response comprehensiveness, therefore, these nanosystems can not precisely respond to multiple signals in the complex physiological environment of a transplanted organ, ultimately reducing therapeutic efficacy and precision 180. The development of multi-stimuli responsive systems should be prioritized, including temporally controlling sequential responses and optimizing designs based on clinical needs to overcome these challenges and advance practical applications. In addition to these bench-scale research-specific limitations, clinical translation of nanomedicine faces a broad set of challenges 176. First, the progression of nanomedicine to clinical phases remains markedly hindered. Clinical translation of nanomedicine in organ transplantation has been demonstrated by the application of ultrasmall superparamagnetic iron oxide particles (USPIOs) in a phase II/III study (NCT02319278). They were employed as an MRI contrast agent for noninvasive diagnosis of myocardial inflammation in patients with cardiac transplant rejection. However, there are very few reports on clinical use of nanomedicine during the transplantation process. A few hurdles must be overcome for clinical translation of nanomedicine. Healthy or single-diseased animals are predominantly employed in preclinical models, and it is challenging to use these animal models to replicate the complex pathological state and the individual variability observed in human organ transplantation 177. The modulation of physicochemical properties of nanoparticles, the regulation of their behavior, and the control of their exposure levels are over-simplified in the current preclinical models. Human organ transplantation involves highly heterogeneous patient populations and complex physiological environments with varied immune systems, resulting in a significantly diminished efficiency of targeted delivery and a remarkably low accumulation level of nanomedicine in the transplantation site. Consequently, the assessment of preclinical toxicity and the prediction of therapeutic efficacy may not be translatable from current preclinical models to humans. To address these translational challenges, primate transplantation models to accurately capture the characteristics of human physiology should be employed during translation studies of nanomedicine for organ transplantation to reduce interspecies differences 178. Additionally, artificial intelligence can integrate nanoparticle characteristics with human physiological parameters to improve the prediction of *in vivo* distribution, metabolism, and clearance, thereby guiding the design of effective nanocarriers for organ transplantation 179 (**Figure 9**).

The toxicity of nanomaterials is another very important issue with the use of nanomedicine in case of transplantations. Despite the methods of nanoparticle delivery eliminating systemic toxicity in relation to the chronic use of immunosuppressants, the nanocarriers can cause cellular damage and tissue-specific toxicity, making them hazardous to the health of the patient. Indicatively, although gold nanoparticles are said to be extremely biocompatible, bovine serum albumin (BSA)-coated spherical gold nanoparticles take long before their clearance in the liver and the spleen as a result of which the macrophage infiltrates and their level of fibronectin increases 180. These results indicate that the long-term toxicity may be triggered by the long-term accumulation of gold nanomaterials and long-term retention in the liver and spleen may result in the development of the possibility of tissue fibrosis. Moreover, the gold nanoparticles have been associated with part of the damage done to the cardiac tissue; prove exists that gold-silver bimetallic nanoparticles would reduce the cardiomyocyte activity significantly 181. Even though iron oxide nanoparticles are approved by FDA to be used in MRI, and have been actively used to monitor posttransplant patients, they potentially possess cardiac toxicity. Recently, it was revealed that ultrafine nanoparticles of Fe_3_O_4_, SiO_2_, and gold provoked the production of ROS in the cardiac tissue. Fe_3_O_4_ nanoparticles were identified to cause the most significant amount of hydroxyl radicals (·OH) that can potentially cause acute heart failure and result in death 182. The main mechanism of action due to which the toxic effect is observed is Fenton-type reactions catalyzed by iron ions, which transform the hydrogen peroxide (H_2_O_2_) into more oxidizing hydroxyl radicals (·OH) thus leading to extreme oxidative damage to the cellular elements. There is therefore, a vital need to undertake thorough evaluation of the effect of physicochemical properties of nanomedicine on the body before the nanomedicine is implemented in an organ transplanting procedure. Furthermore, there is a dire need to closely survey the possible toxicological risks nanomedicine can have on human health in the long run.

Manufacturing and regulatory challenges are other obstacles for the translation of nanomedicine into organ transplantation practices. Owing to the structural complexity of nanomedicine, multi-step and sophisticated manufacturing processes are often needed, while it is challenging to maintain process consistency and reproducibility at scale 183. Unlike conventional chemical pharmaceuticals, nanomedicine does not have a standardized process protocol. Although stringent stability monitoring for chemical pharmaceuticals and nanomedicine is applied during manufacturing and storage, the quality and consistency can vary significantly between batches of nanomedicine, and this challenge may become particularly prominent during scale-up production. The microfluidic technology could prove as a viable technical method of continuous production on a large scale by strictly regulating the size of the nanoparticles, morphology of the particles and other constituents operating within the microfluidic systems below a millimeter diameter. Not only does this method improve batch-to-batch consistency, but also allows real time quality performance during the production process by online monitoring of essential quality parameters 184. At the regulatory tier, the regulatory frameworks that are currently in use cannot be applied to the highly developed nature of nanomedicine. The regulatory bodies are faced with regulatory issues which include the delay in approval of the products and lack of harmonized technical requirements in determining the complex nature of nanomedicine. To overcome these issues, it is important to formulate standards of monitoring that will be used to monitor the key or critical quality attributes of nanomedicine like physicochemical properties, biodistribution. Additionally, the experience of current regulatory circles of organ transplantation (e.g., the one of the Organ Procurement and Transplantation Network) can assist in creating a standard data registry system tailored to nanomedicine that will facilitate information dissemination and the creation of a multipurpose assessment platform 185.

The marginal organs of the donors, which include those of old age, dead donors and organs of individuals with underlying conditions, are usually linked to intense oxidative stress reactions so that they become more vulnerable to IRI during transplantation procedure. This height of vulnerability in turn increases the threats of posttransplant rejection and increases the period of functional recovery 186. In order to address these challenges, implementation of nanomedicine along with the NMP technology are suggested. NMP accomplishes two roles- to mimic physiological properties in keeping the organs alive and to enhance the absorption ability of nanomedicine. As an illustration, Tietjen *et al.* have shown that targeting of renal endothelial cells is accurate provided that anti-CD31 antibodies undergo covalent conjugation to the surfaces of nanoparticles. This can be achieved through the NMP platform to ensure the nanoparticle absorption into the kidneys transplanted is dramatically improved and therefore constitutes a new method of repairing ex vivo donor organ transplants 83. Furthermore, the nanomedicine can be designed to deal with the particular pathologies existing in the organs of donors and hence enhance the quality of organs of marginal sources. As an illustration, nanomedicine to regulate the activities of inflammatory pathways might be optimized to suit donors with fatty liver disease, whereas nanomedicine to regulate the activities of apoptotic pathways might be optimized to suit older donors. These specific intervention measures possess great possibilities in terms of a systematic use of marginal donors and increase in transplant rates of survivals, thus providing enormous possibilities of widening donor base. The ischemia-free transplantation is a technique that is caused by the NMP platform whereby it provides continuity of hemoperfusion during the organ acquisition procedure, preservation, and implantation. The technology is successful in doing away with IRI and curbing the associated problems of traditional transplantation, which is marked by ischemic interruption 187. It has been shown that ischemia-free liver transplantation (IFLT) substantially reduces early graft dysfunction rates postoperatively from 24% for traditional liver transplantation to 6%. IRI is entirely avoided by IFLT and this intervention significantly increases survival rates in marginally donor liver transplant patients 188. The ischemia-free transplantation technology solves and eliminates the IRI, which is a serious hindrance to the quality of the graft, by avoiding the ischemic process. Reparative effects can be increased by specific application of nanomedicine during transplantation. Combination of these strategies would be able to grow the availability of donors considerably and enhance the success rates of transplantations.

Also, nanomedicine can be specifically directed to certain cell organelles such as mitochondria, the endoplasmic reticulum and lysosomes that results in higher drug concentration at the sub cellular level which allows transplant-associated pathological processes to be tightly controlled. Mitochondrial pathology and endoplasmic reticulum-stress are common subcellular pathological changes that are observed in case of immune rejection and post-transplantation IRI. To illustrate, in the case of allogeneic transplantation, TCMR is closely associated with the increase in the endoplasmic reticulum stress of the host lymphocytes, specifically, CD8^+^ T cells. Thus, the endoplasmic reticulum unfolded protein response (UPR) modulation method has become a promising way to alleviate rejection 189. In Shi *et al.*, the nanoparticle system of delivering KIRA6, an upr pathway inhibitor, was optimized through conjugation of the endoplasmic reticulum-targeting peptide pardaxin to LNPs 190. The pardaxin peptide promoted the piling of the LNPs at the endoplasmic reticulum of the lymphocytes hence preventing the UPR sensor function. This mechanism contributed greatly to the reduction of immune rejection and increase of graft survival in the murine model of allogeneic skin transplantation. Besides the endoplasmic reticulum, we can mention nanocarriers targeting the mitochondria that can be useful to address the energy metabolism and reduce the oxidative stress linked to transplanted organs 191. Indicatively, in their study, Yuan *et al.* utilized CoQ10, a type of coenzyme, when delivering to the donor heart mitochondria; the CoQ10 was delivered by a nanosystem based on mitochondrion-targeting (SS31), which targeted the mitochondrion. This nanosystem was able to decrease production of ROS in the posttransplant mitochondria and successfully alleviate IRI 134. Additionally, it is possible to discuss the microenvironmental features of subcellular organelles in order to allow drug-sensitive release. To present an example, a nanomedicine product developed by Shen *et al.* referred to as the anti-CD4 antibody-modified nanomedicine (Tac-NP-CD4Ab) was specific to the CD4^+^ T cells. The aldehyde bonded in its polymer shell was hydrolyzed under acid catalysis within an acidic environment (pH 5.0) of the lysosomes leading to the shell degradation and the release of the drug bound within the shell. This strategy may be applied in high selective drug release and activation inside lysosomes utilizing nanocarriers 148. Through one of the ways is to apply the subcellular level of pathological regulation following organ transplantation and further development of the design of specific nanocarriers to enable a multi-target regulation of the immune rejection and the mitigation of the IRI by means of organelle-targeting specificity, thereby affording to protect the material functionality of the transplanted organ better.

Moreover, the reoccurrence of already existing illnesses among recipients after transplantation is also a significant clinical problem. There are many cases of autoimmune disorder, including autoimmune hepatitis, type 1 diabetes and focal segmental glomerulosclerosis (FSGS), that re-emerges after transplantation, possibly due to the re-awakening of pathogenic autoimmune cells and rise in the level of antibody. For recipients with preexisting fatty liver disease, postoperative dysbiosis of the gut microbiota and portal vein transport of gut-derived inflammatory mediators may lead to recurrent hepatic steatosis or nonspecific hepatitis 192. Additionally, patients with a history of cancer may encounter increased risks of tumor recurrence after they are subjected to immunosuppressive therapy. Collectively, it is critical for disease management following transplantation 193. Nanotechnology may be utilized to develop innovative intervention strategies for the prevention of disease recurrence following transplantation. To address tumor recurrence following liver transplantation under long-term immunosuppression, a nanomedicine (SRNP) was developed for co-delivering RAPA and a camptothecin prodrug, oligoCL₂₈-SN38 194. Esterase-mediated hydrolysis of oligoCL₂₈-SN38 resulted in the release of SN38 within a tumor microenvironment to induce direct tumor cell death. Concurrently, RAPA inhibited mTOR signaling to suppress angiogenesis and cell proliferation of T cells, thus achieving immunomodulation. In a rat liver transplantation model, SRNP treatment significantly prolonged graft survival. In a combined cancer-transplantation model, it simultaneously controlled tumor growth and prevented rejection. This synergistic delivery system provides a pivotal strategy for meeting dual needs of postoperative immunosuppression and antitumor immunity. Moreover, nanocarriers can adeptly modulate posttransplant autoimmune responses through targeted delivery of self-antigens. For example, Jamison *et al.* developed a delivery platform (2.5HIP-PLGA) by employing PLGA nanoparticles enriched with hybrid insulin peptide 2.5HIP, effectively extending the survival of isogeneic islet transplants in mice with type 1 diabetes 195. Upon intravenous administration, 2.5HIP-PLGA particles were sequestered by splenic marginal zone macrophages for antigen presentation, which efficiently induced exhaustion of 2.5HIP-specific effector T cells and promoted the expansion of Tregs, ultimately diminishing the recurrence of posttransplant diabetes. The expansions of this antigen-delivery nanomedicine approach involve directing the delivery of self-antigens and donor-derived HLA antigens into lymphoid organs containing high levels of APCs, including the spleen and liver to induce antigen-specific immune defense which will become more effective in preventing posttransplant rejection and alloimmune responses to prevent recurrence of postoperative diseases.

## 6. Conclusion

There are various novel approaches that have demonstrated better results in the field of organ transplantation by use of nanomedicine. Nanomedicine enhances donor quality during the donor organ preservation phase through in vitro perfusion and preservation injury reduction by even rewarming methods, which helps in eliminating the problem of organ shortage. In an attempt to reduce the IRI in transplantation, nanomedicine exploits stimuli responsiveness and targetability to effectively address the depletion of ROS at ischemic location, amplify the abrogation of inflammatory reactions, and decrease tissue losses. To achieve immune regulation, nanomedicine enhances targetability and bioavailability of immunosuppressive drugs, increasing their efficacy while reducing their systemic side effects. Nanomedicine can also promote Treg expansion by targeting lymphoid organs to establish localized immune tolerance for sustained anti-rejection effects. For real-time monitoring, nanosensors dynamically track the transplanted organ function, noninvasively assess the IRI severity, detect early rejection warning signs, and evaluate drug efficacy. Molecular precision for the diagnosis technique provides technical support for the development of precision nanomedicine. However, there are a few challenges for clinical translation of nanomedicine for organ transplantation, including evaluation of biodistribution and safety of nanomedicine in appropriate animal models or *in vitro* biomimicking organs, and the development of scalable production technology and regulatory frameworks. Future efforts should be devoted to revealing pathological mechanisms of organ transplant-associated diseases, developing novel nanomedicine strategies, expanding application of nanomedicine in the repair of marginal donor organs, achieving precise subcellular delivery, and preventing postoperative disease recurrence. With these efforts, nanomedicine will emerge as a core driver of precision medicine, transforming organ transplantation from the current objective of “successful survival” to a new era of “long-term quality outcomes”, ultimately bringing hopes to patients with end-stage diseases.

## Figures and Tables

**Table 1:** Nanocarrier design and composition in organ transplantation.

NPs	Components of nanocarrier	Material type	Design strategy	Functional unit	Corresponding diseases	Ref
PLGA-FK506-NP	PLGA	Organic	Passive targeting	/	Cardiac allograft acute rejection	62
LNP	SM102, DSPC, cholesterol	Organic	Passive targeting	/	Liver IRI	63
PEG-OligoLys	PEG, oligo(l-lysine)	Organic	Passive targeting	/	/	64
CS-Lys/GP	Chitosan, αβ-glycerophosphate, l-lysine	Organic	Passive targeting	/	/	65
AuPt NPs	Gold, platinum	Inorganic	Passive targeting	/	Kidney IRI	66
PEI-arg@MON@BA	MON	Hybrid	Passive targeting	/	Liver IRI	67
AuNPs-Nanobody	Gold	Inorganic	Active targeting	VHH7	/	68
heparin/CaCO_3_/CaP NPs	Heparin/CaCO_3_/CaP	Hybrid	/	/	/	69
M-NP	Macrophage membrane, PLGA	Hybrid	Active targeting	Toll-like receptor 4	Liver IRI	70
FNVs@RAPA	Extracellular nanovesicles (E. C. grandis, mesenchymal stem cells)	Hybrid	ROS-responsive	Phenylborate ester bond (Azide precursor)	Heart transplant rejection	71
PEGylating 40 nm NPs	PS-COOH particles, PEG	Organic	Passive targeting	/	/	72
TA-FNP	PLGA	Organic	Active targeting	Tannic acid	/	73
PNIPAM-coated nanostructures	PNIPAM, polystyrene	Organic	Passive targeting	/	/	74
FK506 cochleates	PS70, cholesterol	Organic	Passive targeting	/	Heart transplantation rejection	48
aCD3/F/AN	Poly (γ-glutamic acid)	Organic	Active targeting	Anti-CD3e f(ab′)2 fragment	Melanoma	75
GG NP	Polymer gellan gum	Organic	Active targeting	Anti-CD3/CD28	/	76
AuNP	Gold	Inorganic	Active targeting	mannose, galactose, fucose	/	77
Nanovesicles	Membrane (macrophage)	Hybrid	Active targeting	PD-L1/CTLA-4	Skin and heart transplantation rejection	78
DPNP	DHA	Organic	Active targeting	DHA	Heart transplantation rejection	79
HATM	HA, 4-Methoxyphenyl thiourea	Organic	Active targeting/ROS-responsive	HA/TK linkage	Kidney IRI	80
GNP-HClm	Gold	Inorganic	Active targeting	HA	Bronchiolitis obliterans syndrome	81
PEG-dendron	PEG	Organic	*Ex vivo* incubation	/	Islet transplant rejection	82
Polymeric NP	PLA-PEG	Organic	Active targeting	Anti-CD31 antibody		83
PMON@Pt	MON	Hybrid	ROS-responsive	PBAP	Liver IRI	84
HRRAP NP	HA	Organic	PH-responsive	Hydrazone bond	Atherosclerosis	85
LNP	DODAP/gas vesicles	Organic	PH-responsive/ultrasound-responsive	DODAP	Heart transplantation rejection	86
PLG-g-LPEG/TAC	PEG, poly (l-glutamic acid)	Organic	Enzyme-responsive	Gly-Leu	Liver transplantation rejection	87
GBLI-2	/	/	Enzyme-responsive	IEFD	colorectal carcinoma	88
UCNP@mSiO₂@SP-NP-NAP	Mesoporous silica	Inorganic	Light-responsive	Spiropyran, nitrobenzyl group	Heart IRI	89
MN-siRNA	Dextran, iron oxide	Inorganic	Magnetic-responsive	Iron oxide	Islet transplant rejection	90
sIONP	PEG, trimethyl silane, silica, IONP	Inorganic	Magnetic-responsive	Iron oxide	Organ rewarming injury	91

NPs: nanoparticles; DSPC: 1,2-Distearoyl-sn-glycero-3-phosphocholine; PEG: polyethylene glycol; MON: mesoporous organosilica; VHH7: MHCII Antibody; IRI: ischemia reperfusion injury; PLGA: poly (lactic-co-glycolic acid); PNIPAM: poly (*N*-isopropyl acrylamide); PS70: phosphatidylserine; PD-L1: programmed death ligand 1; CTLA-4: cytotoxic T-lymphocyte-associated antigen 4; DHA: docosahexaenoic acid; DODAP: 1,2-Dioleoyl-3-dimethylammonium-propane; HA: hyaluronic acid; IEFD: Ile-Glu-Phe-Asp; IONP: iron oxide nanoparticle. LNP: lipid nanoparticle; PBAP: phenylboronic acid pinacol ester; ROS: reactive oxygen species; TK: thioketal.

**Table 2:** Applications and efficacy of nanomedicines in organ transplantation.

NPs	Material type	Design strategy	Drug	Corresponding disease	Results	Ref
MMF-NP	Organic	*Ex vivo* perfusion	Mycophenola-te mofetil	Heart transplantation rejection	Suppressing early post-transplant inflammation.	133
CoQ10@TNPs	Hybrid	*Ex vivo* perfusion/active targeting	CoQ10	Heart IRI	Significantly suppressing mitochondrial ROS generation and enhancing cardiac function post-transplantation.	134
SPIONs	Inorganic	Magnetic-responsive	/	Organ rewarming injury (heart, islet)	Successfully rewarming the organ (heart, islet) after cryopreservation	135, 136
sIONP	Inorganic	Magnetic-responsive	/	Organ rewarming injury (kidney, liver)	Reducing rewarming injury in cryopreserved organs (kidney, liver) by nanowarming.	137-139
n(SOD-CAT)	Organic	Passive targeting	SOD, CAT	Liver IRI	Suppressing apoptosis and mitigating histopathological damage in IRI-induced liver injury.	140
Cu_us_-pC@MnO₂@PEG	Inorganic	Passive targeting	/	Liver IRI	Effectively suppressing ROS accumulation and concurrently reducing hepatocyte necrosis.	141
BX-001N	Organic	Passive targeting	Synthesize bilirubin 3α	Kidney IRI	Suppressing early post-transplant inflammation.	142
Nano-taurine	Inorganic	Active targeting	Taurine	Liver IRI	Exhibiting superior efficacy and safety over free bilirubin in a renal IRI model.	143
MOFSP	Hybrid	/	Salidroside	Heart IRI	Selectively accumulating in hepatic IRI sites and demonstrating a significant hepatoprotective effect.	144
hUC-MSC-Evs	Organic	Active targeting	Mitochondria	Liver IRI	Providing localized, sustained drug release and thereby alleviating cardiac IRI.	145
NP-Ly6G(2-DG)	Organic	Passive targeting	2-DG	Liver IRI	Restoring the neutrophil mitochondrial function and ultimately alleviating liver IRI	146
LNPs-sirt4 mRNA	Organic	Passive targeting	SIRT4 mRNA	Liver IRI	Mitigating pulmonary IRI by suppressing neutrophil glycolysis.	63
aNP	Organic	Active targeting	Tacrolimus	Skin transplantation rejection	Suppressing ferroptosis and consequently alleviating hepatocyte death.	147
Tac-NP-CD4Ab	Inorganic	pH-responsive	Tacrolimus	Kidney transplantation rejection	Effectively preventing rejection and mitigating nephrotoxicity risk of tacrolimus.	148
Ce6-NP-MCP-1	Organic	Active targeting/Ultrasound-responsive	Ce6	Heart transplantation rejection	Inhibiting B cell plasmacyte differentiation and DSA production and reducing conventional drug nephrotoxicity.	149
AU-siRetn	Inorganic	Passive targeting	siRetn	Liver transplantation rejection	Depleting macrophages, reducing inflammatory cell infiltration in the cardiac allograft, and prolonging recipient survival via sonodynamic therapy.	150
C5 siRNA-LNP	Organic	Passive targeting	C5 siRNA	Kidney transplantation rejection (ABMR)	Reducing inflammatory cell infiltration and T cell over-proliferation in the liver, and ameliorating the transplant liver function.	151
rPS	Organic	Passive targeting	RAPA	Islet transplantation rejection	Blocking excessive activation of the complement pathway, significantly prolonging graft survival, and improving the renal function.	152
FasL@Rapa NPs	Organic	Active targeting	RAPA	Islet transplantation rejection	Significantly suppressing T cell proliferative responses to donor antigens and maintaining normal blood glucose levels for 100 days in islet-transplanted mice.	153
BEZ235@NP	Organic	Passive targeting	BEZ235	Heart transplantation rejection	Effectively suppressing CD8⁺ T cell proliferation, promoting T_reg_ expansion, and prolonging transplanted islet survival.	154
FTY720@TNP	Hybrid	Passive targeting	FTY720	Heart transplantation rejection	Prolonging the survival of mouse cardiac allografts, reducing the activation and infiltration of CD4⁺ and CD8⁺ T cells, and increasing the T_reg_ proportion.	155
MECA-79-anti-CD40L-NP	Organic	Active targeting	/	Heart transplantation rejection	Increasing the local T_reg_/T_eff_ ratio in lymph nodes and thereby promoting long-term graft survival.	156
KSINPs	Inorganic	/	/	Islet transplantation rejection	Prolonging the survival of heart grafts to 80 days.	157
GBRNs	Inorganic	Enzyme-responsive	/	Heart transplantation rejection	Remodeling the splenic immune environment, and enabling long-term survival and function of transplanted islets.	158
ErGZ	Inorganic	Enzyme-responsive	/	Skin and islet transplantation rejection	Enabling early detection of rejection by GBRNs prior to significant functional loss in cardiac allografts.	159
MTBPB/GPs	Organic	ROS-responsive	/	Skin transplantation rejection	Enabling real-time monitoring of intragraft granzyme B activity with high sensitivity and specificity.	160
DIPT-ICF	Organic	/	/	Kidney transplantation complications	Enabling early detection of acute rejection and evaluation of immunosuppressive therapy efficacy.	161
APN_SO_	Organic	ROS-responsive	/	Liver injury	Achieving clear images of the vascular structure of the transplanted kidney, which can be used to assess the patency of the postoperative urinary tract anastomosis, vascular stenosis, and the degree of IRI	162
MN-siRNA	Inorganic	Magnetic-responsive	/	Islet transplantation rejection	Non-invasively assessing the severity of hepatic IRI by combining real-time *in vivo* fluorescence imaging with urine fluorescence intensity quantification.	90

ABMR: antibody mediated rejection. CAT: catalase; IRI: ischemia reperfusion injury; NPs: nanoparticles; RAPA: rapamycin; ROS: reactive oxygen species; SOD: superoxide dismutase.
